# Patient-reported outcomes in integrated health and social care: A scoping review

**DOI:** 10.1177/20542704241232866

**Published:** 2024-03-24

**Authors:** Sarah E Hughes, Olalekan L Aiyegbusi, Christel McMullan, Grace M Turner, Nicola Anderson, Samantha Cruz Rivera, Philip Collis, Jon Glasby, Daniel Lasserson, Melanie Calvert

**Affiliations:** 1 Centre for Patient Reported Outcomes Research, Institute of Applied Health Research, 1724 University of Birmingham, Birmingham, UK; 2 National Institute for Health and Care Research (NIHR) Applied Research Collaboration West Midlands, Birmingham, UK; 3 Birmingham Health Partners Centre for Regulatory Science and Innovation, 1724 University of Birmingham, Birmingham, UK; 4 National Institute for Health and Care Research (NIHR) Blood and Transplant Research Unit in Precision Cellular Therapeutics, 1724 University of Birmingham, Birmingham, UK; 5 National Institute for Health and Care Research (NIHR) Birmingham Biomedical Research Centre, University of Birmingham and University Hospitals Birmingham NHS Foundation Trust, Birmingham, UK; 6 National Institute for Health and Care Research (NIHR) Surgical Reconstruction and Microbiology Centre, University of Birmingham and University Hospitals Birmingham NHS Foundation Trust, Birmingham, UK; 7 Warwick Medical School, University of Warwick, Coventry, UK; 8 DEMAND Hub, 1724 University of Birmingham, Birmingham, UK; 9 Patient partner, Birmingham, UK; 10 School of Social Policy, 1724 University of Birmingham, Birmingham, UK; 11 IMPACT (Improving Adult Social Care Together), 1724 University of Birmingham, Birmingham, UK; 12 Department of Geriatric Medicine, Oxford University Hospitals NHS Foundation Trust, Oxford, UK

**Keywords:** health service research, non-clinical, long-term care, geriatric medicine, clinical, other statistics and research methods, statistics and research methods, non-clinical, health informatics, non-clinical

## Abstract

**Background:**

Patient-reported outcomes (PROs) have potential to support integrated health and social care research and practice; however, evidence of their utilisation has not been synthesised.

**Objective:**

To identify PRO measures utilised in integrated care and adult social care research and practice and to chart the evidence of implementation factors influencing their uptake.

**Design:**

Scoping review of peer-reviewed literature.

**Data sources:**

Six databases (01 January 2010 to 19 May 2023).

**Study selection:**

Articles reporting PRO use with adults (18+ years) in integrated care or social care settings.

**Review methods:**

We screened articles against pre-specified eligibility criteria; 36 studies (23%) were extracted in duplicate for verification. We summarised the data using thematic analysis and descriptive statistics.

**Results:**

We identified 159 articles reporting on 216 PRO measures deployed in a social care or integrated care setting. Most articles used PRO measures as research tools. Eight (5.0%) articles used PRO measures as an intervention. Articles focused on community-dwelling participants (35.8%) or long-term care home residents (23.9%), with three articles (1.9%) focussing on integrated care settings. Stakeholders viewed PROs as feasible and acceptable, with benefits for care planning, health and wellbeing monitoring as well as quality assurance. Patient-reported outcome measure selection, administration and PRO data management were perceived implementation barriers.

**Conclusion:**

This scoping review showed increasing utilisation of PROs in adult social care and integrated care. Further research is needed to optimise PROs for care planning, design effective training resources and develop policies and service delivery models that prioritise secure, ethical management of PRO data.

## Introduction

Recent years have witnessed significant progress in the delivery of integrated health and social care.^1^ The move towards integrated care has been motivated by an ageing population with multiple chronic conditions, fragmented and costly care systems, a focus on patient-centred care and empowerment and recognition by service providers of the need for a more joined-up approach to care.^2^ Integrated health and social care aims to provide comprehensive and seamless services to individuals through the coordination and collaboration of healthcare and social care services to deliver holistic support.^3^ Patient-reported outcomes (PROs), as self-reported measures of how an individual feels and functions, have the potential to play a crucial role in integrated care and adult social care settings.^4,5^ Patient-reported outcomes provide a means of capturing the unique perspectives of individuals receiving care, enabling them to actively participate in their own care journey. Patient-reported outcomes can bridge the gap between healthcare and social care sectors by capturing shared outcomes related to physical health, mental wellbeing, social functioning and quality of life. By capturing feedback on various dimensions of wellbeing, PROs allow for tailored care plans that meet individuals’ specific needs and goals, enabling more effective, person-centred care.^6^ Patient-reported outcomes also support the evaluation of care interventions, providing feedback and identifying areas for improvement.^7^ Patient-reported outcomes can promote equity and inclusion, ensuring care services are responsive to the unique needs of individuals and fostering partnership and collaboration, encouraging shared decision-making and giving care recipients a sense of ownership over the care process.^8,9^ Alignment of PROs with person-centred care, their capacity to capture comprehensive outcomes, and to support service evaluation highlight their potential to shape the delivery of care and support. Despite these potential benefits, current utilisation of PROs in the context of integrated care and adult social care is unclear. We aimed to conduct a scoping review to identify PRO measures used in integrated care and social care settings and to chart the evidence relating to their use, including barriers and facilitators influencing their uptake.

## Methods

This review protocol was published in *BMJ Open*.^10^ Results are reported in accordance with the PRISMA guidelines for scoping reviews.^11^

### Data sources and searches

We searched the following databases from 01 January 2010 to 19 May 2023: Medline (Ovid), Embase (Ovid), PsychInfo (Ovid), HMIC (Ovid), Social Care Online (SCIE), ASSIA (Pro Quest) and Web of Science. The authors developed the search strategy (Supplementary Appendix 1) with input from University of Birmingham Information Specialists. We imported search results to Endnote (Version 9.3.3, www.endnote.com) reference management software for screening.

### Study selection

Articles were included if published in English and described studies of any design that reported use of PROs and involved adults (18+ years) who were direct users of integrated care or social care services. We excluded articles reporting only carer-reported or proxy-reported outcome measures and articles reporting studies undertaken with no social care or integrated care involvement. Two researchers (from SEH, CM, GMT, NA, SCR) independently screened articles against the eligibility criteria. We screened titles and abstracts followed by review of full-text articles. Discussion and involvement of a third reviewer resolved disagreements. Eligibility criteria were refined iteratively until we achieved a minimum threshold for inter-rater agreement of 75%.

### Data extraction

#### Piloting/calibration exercise

A data charting form was developed and piloted on 25 included articles to ensure data capture consistent with review objectives.

#### Data charting process

Included articles were imported into Covidence software (www.covidence.com) for data charting. Thirty-six articles (22.6%) were charted in duplicate by two reviewers independently (from SEH, CM, GMT, NA, SCR) to ensure data were captured consistently by reviewers.^12^ Consistent with scoping review methodology, we charted data as reported in the included sources. Study authors were not contacted, and missing information was entered in the data charting form as ‘unclear’ or ‘not reported’. We extracted the following data from all included articles: bibliometric characteristics, study design, study population, setting, PRO measure characteristics, PRO measure use as an intervention, client and system level outcomes as well as implementation barriers and facilitators.

#### Data synthesis and analysis

Evidence was mapped using descriptive statistics to report bibliometric characteristics of the included articles, study design, population, setting and PRO characteristics. We utilised thematic analysis and the Framework method to synthesise information on the implementation of PROs, coding line-by-line the results sections of any article for which data were extracted during the charting process to the following categories: (1) use of PRO measures as an intervention, (2) client-level outcomes for PRO intervention; (3) system-level outcomes; (4) barriers to PRO implementation and (5) enablers of PRO implementation.^13,14^ Inductive coding captured additional concepts identified in the data.^12^ Quality appraisal, optional in a scoping review, was not undertaken.^11^

#### Lived experience involvement and engagement

People with lived experience of care and support reviewed the study protocol, provided recommendations for search strategy development and reviewed study findings.

## Results

**Figure 1.**
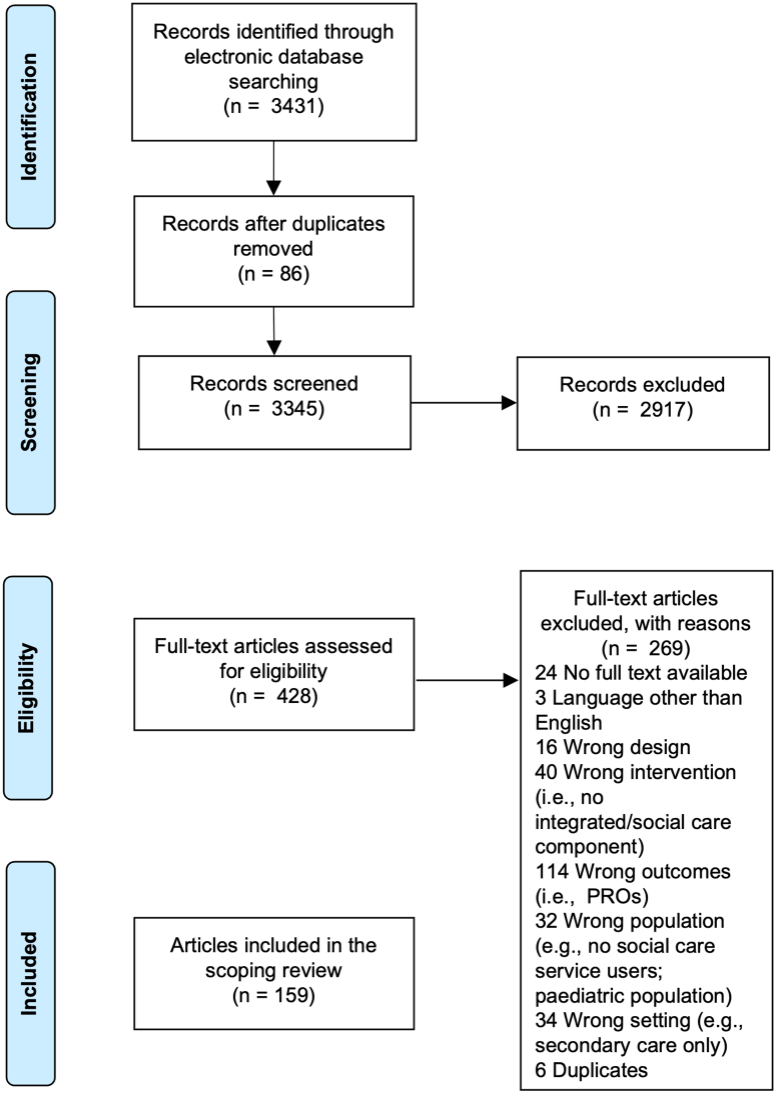
PRISMA flow diagram showing the study selection process.

Searches identified 3431 records and 159 were eligible for inclusion (Figure 1).

### Study characteristics

Eligible articles (Supplementary Appendix 2) were published between January 2010 and May 2023. The number of published articles increased over time (range = 0–22 articles per year) with numbers declining during the COVID-19 pandemic (Supplementary Appendix 3). Datasets (*n* = 166) from the studies reported in the 159 articles were from 39 countries, with most articles originating in the United Kingdom (*n* = 38, 22.9%) or United States (*n* = 34, 20.5%). When grouped by region, most originated from Europe (*n* = 76 articles, 47.8%). The most frequently reported study designs were cross-sectional studies (*n* = 44, 27.7%), psychometric studies (*n* = 22, 13.8%), randomized control trials (*n* = 19, 11.9%) and cohort studies (*n* = 19, 11.9%) (Table 1). Mean sample size was 880 (median = 192, range = 5–29,935; available for 153 studies in 149 articles).

**Table 1.** Study designs reported in the included articles (*n* = 159).

Study design	*n*	%
Cross-sectional study	44	27.7%
Psychometric study	22	13.8%
Randomized controlled trial	19	11.9%
Cohort study	19	11.9%
Non-randomised experimental study	15	9.4%
Mixed Methods	9	5.7%
Qualitative research	8	5.0%
Pilot study	8	5.0%
Review article	7	4.4%
Economic evaluation	4	2.5%
Text and opinion	2	1.3%
Case series	1	0.6%
Delphi Study	1	0.6%

### Population characteristics

Participants’ mean age was 72.8 years (median = 77 years, range 35.7–87.6 years; *n* = 117 articles), and mean percentage of females was 62.1% (range = 23.4–100; SD = 14.8, 147 articles). Approximately half of the articles (*n* = 87, 54.7%) involved older adults. The definition of ‘older adult’ varied, with 35 articles (22.0%) defining older adults as aged 65+ years. 49 (31.8%) of 154 eligible articles reported participants’ ethnicity or race. Pooling of ethnicity data across included articles was not possible due to reporting variability. Study samples involving specific groups (e.g. adults with intellectual disabilities, adults experiencing homelessness) were younger. Article authors frequently defined the population of interest by service use or place of care (e.g. nursing home residents, recipients of home care services). We were unable to pool co-morbidity data due to article heterogeneity.

### Setting characteristics

Participants were community-dwelling in 57 articles (35.8%), residents of long-term care homes (i.e. nursing homes, residential care homes) in 38 (23.9%) articles and recipients of supported living (e.g. independent living and retirement communities) in 9 (5.7%) articles. Five studies (3.1%) were conducted in the context of social care day services. Participants accessed homeless services in five (3.1%) articles, palliative care services in 11 (6.9%) articles and integrated health and social care services in 3 (1.9%) articles (Table 2).

**Table 2.** Characteristics of the settings targeted in the included articles (*n* = 159).

Target setting	Articles	%
Community-dwelling (i.e. own home)	57	35.8%
Long-term care home (i.e. nursing home)	38	23.9%
Multiple care settings	30	18.9%
Palliative care	11	6.9%
Supported living (i.e. independent living facility, retirement community)	9	5.7%
Day services	5	3.1%
Homeless services	5	3.1%
Integrated care services	3	1.9%
Not specified	1	0.6%

### Patient-reported outcome characteristics

Patient-reported outcome measures (*n* = 216, Supplementary Appendix 4) were extracted from the included articles. In 157 available articles (text/opinion articles were excluded, *n* = 2), the median number of PRO measures reported per article was two (range = 1–13 PROs). Most PRO measures (*n* = 165, 75.7%) were reported in one article, with a median of one article per PRO measure (range = 1–28 articles). Among the most frequently reported PRO measures, the EQ-5D was reported in 28 articles (17.8%), the Geriatric Depression Scales (GDS) was reported in 26 articles (16.6%) and the Adult Social Care Outcomes Toolkit (ASCOT) reported in 25 articles (15.9%). The Patient Health Questionnaire-9 (PHQ-9) appeared in 12 articles (7.6%), and the SF-36 reported in eight articles (5.1%).

### Construct coverage

Patient-reported outcomes reported in the included articles were extracted and classified into four construct categories based on classifications proposed by McGilton *et al.*: functional status, psychological status, health status/symptom burden and quality of life (Table 3 and Supplementary Appendix 5).^15^ Most PRO measures identified from the included articles targeted psychological or social constructs/issues (*n* = 121, 56.0%). Nine PRO measures classified as ‘other’ (4.2%) measured aspects of service provision including health literacy, health needs assessment and care planning.

**Table 3.** PRO measures (*n* = 216) classified by outcome construct category.

General category of outcome	*n*	%
Functional status	35	16.2%
Psychological status	121	56.0%
Health Status/Symptom Burden	15	6.9%
Quality of life	36	16.7%
Other	9	4.2%

#### Functional status

Functional status included PRO measures assessing a person's ability to perform activities of daily living.^16^ Thirty-five (16.2%) measures assessed functional status, reported in 43 (27.4%) of 157 available articles. The most frequently reported PRO measures were the Barthel Index (*n* = 7 articles, 4.5% of available articles), and the Index of Activities and Daily Limitations (*n* = 6 articles, 3.8% of available articles).

#### Psychological status

The psychological status category included PRO measures assessing cognitive status, psychological constructs (e.g. anxiety) or social constructs and issues (e.g. loneliness, participation); 121 (56.0%) PRO measures were reported in 86 (54.8%) of the 157 available articles. The most frequently reported measures were the GDS (*n* = 26 articles, 16.6%), the PHQ-9 (*n* = 12 articles, 7.6%), the Multi-dimensional Scale of Perceived Social Support (*n* = 7 articles, 4.5%) and Centre for Epidemiologic Studies Depression Scale (*n* = 7 articles, 4.5%). The ICECAP-O, a PRO measure of capability wellbeing for older people, was reported in five articles (3.2%).

#### Health status/symptom burden

Patient-reported outcomes measures of health-related constructs were included in this category; 22 (14.0%) articles reported symptom burden using 15 (6.9%) different PRO measures. The Edmonton Symptom Assessment System was reported most frequently (*n* = 6 articles, 3.8%) followed by the Patient-Reported Outcomes Measurement Information System (PROMIS) (*n* = 5 articles, 3.2%).

#### Quality of life

About 36 (16.7%) PRO measures assessed quality of life in 98 (62.4%) articles. The EQ-5D, a generic measure of health-related quality of life, was used most frequently (*n* = 28 articles, 17.8%). The ASCOT assessed social-care-related quality of life in 25 articles (15.9%).

### Patient-reported outcome data capture and context of use

About 25 (15.7%) articles utilised paper PRO measures, 11 (6.9%) articles used PRO measures delivered electronically (ePROs), one (0.6%) article used a computer adaptive test (i.e. a PRO measure that tailors the questions based on a respondent's answers) and four articles (2.5%) reported use of multiple formats; 114 articles (71.7%) did not report the format of PRO data capture.

Interviews were used to administer PRO measures in 57 (35.8%) articles and self-completion by participants was reported in 28 (17.6%) articles; 22 articles (13.8%) used multiple administration methods while 48 (30.2%) articles did not report method of administration. Commentary articles and articles reporting secondary data analyses (*n* = 4, 2.5%) were categorised as ‘not applicable’.

Patient-reported outcome measures evaluated intervention benefit in 52 articles (32.7%). Interventions were typically health-focussed therapies delivered in adult social care settings to care recipients. Patient-reported outcome measures were used to collect observational data in 65 articles (40.9%). Eight articles (5.0%) were evaluations of interventions that included PROs as a component of the intervention and 34 articles (21.4%) reported on PRO measure development, adaptation and/or validation.

### Patient-reported outcome implementation

**Figure 2.**
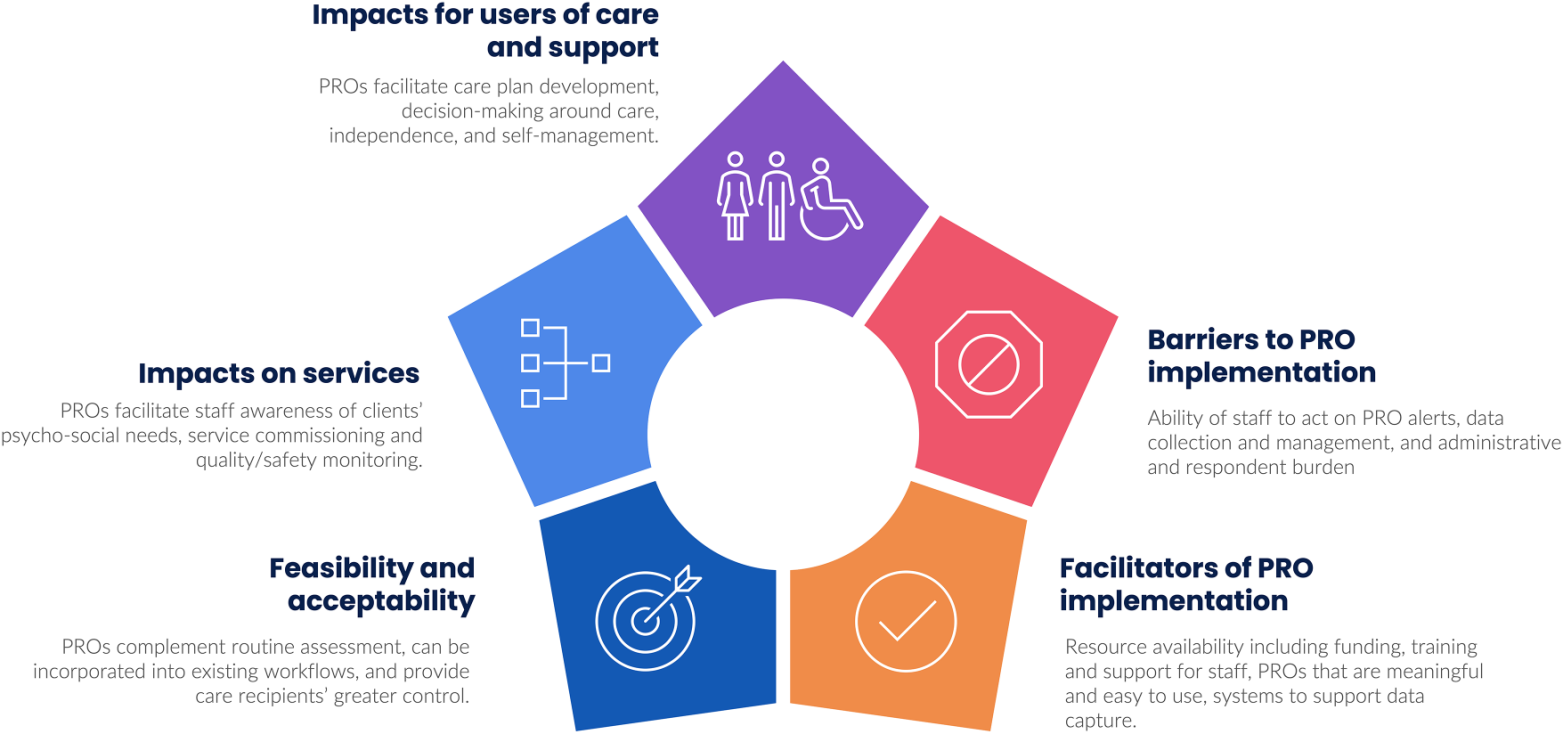
Summary of narrative synthesis findings showing key themes relating to the uptake of PRO measures in integrated care and adult social care.

About 17 (10.6%) included articles were identified from data charting as having data relating to PRO implementation. Thematic analysis of the results sections of these articles yielded five themes: (1) impacts of PROs on users of care and support; (2) system/service-level impacts; (3) barriers to implementation; (4) facilitators of implementation; and (5) feasibility and acceptability of PROs in integrated and adult social care (Figure 2). A narrative synthesis of findings is presented per theme.

#### Impact of PROs on people who use care and support services

About 12 (70.6%) of the 17 articles described potential benefits and disadvantages of PROs for users of integrated or social care services. Patient-reported outcomes were viewed positively for care planning. A Delphi review of an integrated participant assessment system for Adult Day Services (ADS) suggested PROs can assist individual care planning, support funding requests, validate caregiver experiences and assure payers of savings.^17^ Five articles considered PROs advantageous in care plan development and for understanding the links between quality of life and factors that influence it such as health, level of need and the importance and availability of social support.^18–22^ Patient-reported outcomes were also viewed positively as means of facilitating person-centred care^21,23^ by improving communication between care recipients and their care team, increasing user involvement in decision-making and helping prepare for medical appointments.^22,24,25^ Patient-reported outcomes were understood to have potential to support self-monitoring (especially for frail older people and people with multiple, long-term conditions) thereby enabling people to maintain independence in their homes and communities as long as possible.^25,26^ Patient-reported outcome measures were proposed to have positive impacts in terms of their ability to generate alerts to changes of care recipients’ health status^26,27^ and, in the case of community aged care assessments in Australia, to raise awareness of recipients’ psychosocial needs.^21,24^ Monitoring health and wellbeing using PROs was viewed as helpful in supporting ageing populations to live at home.^26^ Potential negative impacts included burden associated with PRO measure completion and potential for PRO measures to highlight negative emotions or distressing circumstances to care recipients..^17,21^ Use of closed-ended questions to measure PROs was considered to privilege measurement over what matters most for care recipients.^17,23^

#### Impacts of PROs on services

In the context of developing a minimum data set for UK adult care homes, PROs, by measuring concepts of importance to residents, were deemed to have potential to support service commissioning and monitoring of care quality, safety and effectiveness.^28^ The use of PROs in benchmarking and quality improvement initiatives was highlighted as important in the Australian aged care sector.^29^ In England, PROs were used in productivity analyses of adult social care through calculation of ‘quality adjusted’ output using indicators drawn from the ASCOT and the annual Adult Social Care Survey.^30^

#### Barriers to PRO implementation

Perceived barriers included limited ability of care staff to act on alerts triggered by PRO scores and a need for training to enable staff to generate timely, appropriate referrals from PRO data.^21,24,26,27^ Data capture and management were also identified as implementation barriers. A pilot study of a nurse-led palliative care intervention in rural Canada identified challenges associated with missing data and longitudinal collection of PROs.^22^ In a study describing adaptation of the ASCOT for use in care homes, stakeholder consultation revealed that how and by whom ASCOT data are collected affected subsequent use and perceived trustworthiness of the data.^31^ Lack of consistency, heterogeneous instruments, absent infrastructure and siloed care systems were identified as barriers to PRO data accuracy.^17,28^ Administrative and respondent burden (i.e. on individuals, family members, carers and practitioners) were viewed as barriers to PRO measure use, with time constraints and measure complexity identified as specific obstacles.^25^ Relevance of PROs for care recipients and payers was highlighted as a potential barrier by a Delphi panel convened to support the development of an integrated participant assessment system for adult day services.^17^

A dearth of PRO measures validated for use in integrated and social care contexts was identified as a further implementation barrier.^23^ Noting PROs’ origins in healthcare, a review of social care occupational therapy services suggested the applicability of existing PRO measures for integrated and social care cannot be presumed and measures designed specifically for these settings may be required.^23^ Accessibility of PRO measures for care recipients was a further concern.^27^ For example, articles describing the development of the ASCOT Easy Read version highlighted potential accessibility and inclusivity barriers such as low health literacy.^27,32^ An Australian study of aged-care services considered it important for PRO data to be valid appraisals of the impact of care and support services on the health, wellbeing and quality of life of care recipients.^18,22^ Lastly, reconciling the requirement for broadly applicable measures with the necessity for an individualised approach to capturing PRO data was considered a further challenge to implementation.^17^

#### Facilitators of PRO implementation

Easy-to-use PRO measures that support longitudinal data collection and PRO data capture systems that encourage information sharing and reduce administrative burden were perceived facilitators of their use. Four articles suggested successful PRO implementation would need to give staff more time for direct care activities.^17,23,28,33^ Further enablers identified from six articles included: (1) appropriate funding, (2) stakeholder buy-in, (3) training for new employees and managers and (4) having procedures for quality assurance.^17,18,26,27,31,32^ Having access to PRO measures that promote individualised care and support was a key consideration in five articles.^18,22,23,25,28^ Lastly, use of accessible measures and sensitive use of proxy-report (particularly for people experiencing cognitive impairment) were identified as key to successful and inclusive PRO implementation.^20,34^

#### Feasibility and acceptability of PROs

PROs were considered to be acceptable and feasible when used for monitoring and as a feedback intervention, did not disrupt routine workloads, and enabled practitioners to identify when additional support may be required, particularly in situations where needs (e.g. loneliness) were not easily observed.^19,20,21,27,31^ Recipients of care and support suggested PROs contributed to a sense of control over their health and wellbeing. PRO measures of social engagement were found to support caseload management and aid policy and managerial decision-making in large-scale Australian aged care organisations.^24,33^

## Discussion

In this study, we carried out a scoping review of the published evidence to explore use of PROs in integrated care and adult social care research and practice. To our knowledge, this review is the first study to provide a broad overview and synthesis of the literature on the use of PROs in these contexts. Excluding the period of the COVID-19 pandemic, the number of articles published per year with an integrated or adult social care focus that reported PROs increased from 2010 to 2023. This trend could be indicative of a move by policy-makers towards integrated care systems, prioritisation of personalisation and a quality-of-life outcomes-based approach to the delivery of care and support.^2,35^

We conducted a thematic analysis to describe potential impacts of PROs on integrated and social care including implementation barriers and facilitators. The review findings relating to the benefits and implementation of PROs were broadly consistent with the published literature describing PRO implementation in healthcare settings. Where PROs were found to promote communication between social care practitioners and people drawing on care and support, PROs have been found to similarly provide healthcare professionals with a structured method to document a patient's problems and to empower patients to discuss issues and concerns of importance with their healthcare team.^9^ Training for time-pressed social care professionals in the administration, interpretation and potential benefits of PROs was another commonly reported barrier consistent with the health literature.^36–38^ Lastly, data management and electronic data capture systems were reported in several studies as not only barriers but also potential facilitators of PRO implementation in integrated care and adult social care. Several articles endorsed the benefits of PRO integration with electronic records, whilst others raised concerns that standardisation and electronic integration may limit personalisation and be a barrier to achieving equity and inclusion. These findings align with trends reported in the published literature on the use of electronic and remote PRO data capture in clinical trials and routine healthcare settings.^39–42^ Inconsistent or non-reporting of ethnicity data in the included articles suggested issues with cultural validity and the inclusive, equitable and ethical use of PROs.^40,43^

### Strengths and limitations

We conducted a broad, comprehensive search of multiple databases and utilised the PRISMA guidance for scoping reviews.^11^ However, we restricted the review to articles published in English and did not undertake quality appraisal. The latter are potential sources of bias although it should be noted that quality appraisal is not required for scoping reviews. Study heterogeneity and a lack of consistency in the concepts, definitions and terminology used across the integrated care and social care literature, likely arising from fragmentation of service delivery and heterogeneous models of care at regional, national and international levels, made it difficult to ascertain article relevance. We addressed this challenge by using a team of reviewers for screening, consensus discussions and iterative piloting of eligibility criteria until inter-rater agreement met a pre-specified threshold.^11^

### Implications for research, policy and practice

The review findings suggest there is currently substantial inconsistency in PRO use across integrated and social care settings, with numerous and diverse PRO measures used primarily as research tools. This finding emphasises the importance of conducting further research to establish a common set of PROs for integrated and adult social care research and practice, acknowledging that additional challenges, such as underfunding, workforce pressures and fragmented care systems, are likely to pose further barriers to their implementation.^44^ A standardised set of PROs could facilitate a more holistic and equitable approach to care delivery. Core outcome sets already exist for a number of populations likely to be in receipt of care and support (e.g. people living with dementia) and a recent mixed-methods study has explored the feasibility of a core outcome set for adult social care.^45,46^ Notably, evidence relating to the challenges encountered in integrating PROs as an element of care and support, as found through this review, highlights the necessity for research to establish best practice guidance for the following: (1) integration of PROs into care planning and delivery; (2) effective training for practitioners to utilise PROs proficiently; and (3) formulation of policies that prioritise secure and ethical management of PRO data. Lastly, carer-reported outcomes were not a focus for this review; however, carers are regarded as co-clients in the social care policy landscape.^35^ Efforts are required to better understand how carer-focused outcome measures are applied in the carer–care recipient relationship and how they are utilised in relation to PRO measures completed by care recipients. The identification and implementation of measures which capture carer outcomes alongside care-recipient outcomes is recommended to ensure the voices of all individuals impacted by care and support are at the heart of policy and service delivery.^35^

## Conclusion

This review charted the peer-reviewed evidence for the use of PROs in integrated health and social care. We concluded that although PROs are used as research tools in integrated and adult social care, there is a need for greater coherence. In the context of service delivery, we found limited evidence of implementation, suggesting PROs are currently underutilised. Considerable work focusing on the routine use of PROs across integrated care and social care services is required if their benefits are to be realised by key groups, most importantly, by those individuals receiving care and support, their families and carers.

## Supplemental Material

sj-pdf-1-shr-10.1177_20542704241232866 - Supplemental material for Patient-reported outcomes in integrated health and social care: A scoping review Supplemental material, sj-pdf-1-shr-10.1177_20542704241232866 for Patient-reported outcomes in integrated health and social care: A scoping review by Sarah E Hughes, Olalekan L Aiyegbusi, Christel McMullan, Grace M Turner, Nicola Anderson, Samantha Cruz Rivera, Philip Collis and Jon Glasby, Daniel Lasserson, Melanie Calvert in JRSM Open

sj-pdf-2-shr-10.1177_20542704241232866 - Supplemental material for Patient-reported outcomes in integrated health and social care: A scoping review Supplemental material, sj-pdf-2-shr-10.1177_20542704241232866 for Patient-reported outcomes in integrated health and social care: A scoping review by Sarah E Hughes, Olalekan L Aiyegbusi, Christel McMullan, Grace M Turner, Nicola Anderson, Samantha Cruz Rivera, Philip Collis and Jon Glasby, Daniel Lasserson, Melanie Calvert in JRSM Open

sj-pdf-3-shr-10.1177_20542704241232866 - Supplemental material for Patient-reported outcomes in integrated health and social care: A scoping review Supplemental material, sj-pdf-3-shr-10.1177_20542704241232866 for Patient-reported outcomes in integrated health and social care: A scoping review by Sarah E Hughes, Olalekan L Aiyegbusi, Christel McMullan, Grace M Turner, Nicola Anderson, Samantha Cruz Rivera, Philip Collis and Jon Glasby, Daniel Lasserson, Melanie Calvert in JRSM Open

sj-pdf-4-shr-10.1177_20542704241232866 - Supplemental material for Patient-reported outcomes in integrated health and social care: A scoping review Supplemental material, sj-pdf-4-shr-10.1177_20542704241232866 for Patient-reported outcomes in integrated health and social care: A scoping review by Sarah E Hughes, Olalekan L Aiyegbusi, Christel McMullan, Grace M Turner, Nicola Anderson, Samantha Cruz Rivera, Philip Collis and Jon Glasby, Daniel Lasserson, Melanie Calvert in JRSM Open

sj-pdf-5-shr-10.1177_20542704241232866 - Supplemental material for Patient-reported outcomes in integrated health and social care: A scoping review Supplemental material, sj-pdf-5-shr-10.1177_20542704241232866 for Patient-reported outcomes in integrated health and social care: A scoping review by Sarah E Hughes, Olalekan L Aiyegbusi, Christel McMullan, Grace M Turner, Nicola Anderson, Samantha Cruz Rivera, Philip Collis and Jon Glasby, Daniel Lasserson, Melanie Calvert in JRSM Open

sj-pdf-6-shr-10.1177_20542704241232866 - Supplemental material for Patient-reported outcomes in integrated health and social care: A scoping review Supplemental material, sj-pdf-6-shr-10.1177_20542704241232866 for Patient-reported outcomes in integrated health and social care: A scoping review by Sarah E Hughes, Olalekan L Aiyegbusi, Christel McMullan, Grace M Turner, Nicola Anderson, Samantha Cruz Rivera, Philip Collis and Jon Glasby, Daniel Lasserson, Melanie Calvert in JRSM Open
