# Supplementary material for: Patient-reported outcomes in integrated health and social care: A scoping review
Source: JRSM Open. 2024 Mar 24;15(3):20542704241232866. doi: 10.1177/20542704241232866 (PMC10962043; doi:10.1177/20542704241232866)
Supplement: sj-pdf-1-shr-10.1177_20542704241232866 - Supplemental material for Patient-reported outcomes in integrated health and social care: A scoping review [file sj-pdf-1-shr-10.1177_20542704241232866.pdf]

S1 Data. Exemplar search strategy with yields

Database: Medline (OVID)
Date of Search: 29-Sep-2020
Note: Lines 48-72 are based on the modified Oxford PROM filter and the search strategy.[1]

	Search string	Records returned
	**Setting**	
1	respite care/	1026
2	respite care.ti,ab.	724
3	hospice/ or home care/ or palliative care/ or terminally ill patients/	89624
4	exp client transfer/ or exp facility discharge/ or exp hospital discharge/ or exp patient selection/ or exp professional referral/ or exp psychiatric hospital discharge/ or exp treatment refusal/ or exp treatment termination/	77245
5	patient transfer.ti,ab.	854
6	hospice.ti,ab.	11409
7	residential home.ti,ab.	321
8	care home*.ti,ab.	3877
9	aged care home*.ti,ab.	94
10	nursing homes/	34874
11	retirement home*.ti,ab.	269
12	adult day care centre*.ti,ab.	15
13	homes for the aged.ti,ab.	524
14	self-care skills/ or aging in place/ or independent living programs/ or rehabilitation/	24336
15	independent living.ti,ab.	2636
16	personalised budget*.ti,ab.	5
17	reablement.ti,ab.	79
18	home nursing.ti,ab.	1323
	**Population**	
19	aged.ti,ab.	564640
20	geriatric patients/	0
21	old* person.ti,ab.	1542
22	(elderly or geriatric).ti,ab.	268074
23	exp Disabilities/ or exp Intellectual Development Disorder/ or exp Developmental Disabilities/	113122
24	disabled person*.ti,ab.	1574
25	disabled people.ti,ab.	1488
26	(disabled adj3 person*).ti,ab.	1845
27	vulnerable population*.ti,ab.	9786
28	vulnerable person*.ti,ab.	300
29	intellectual development disorder/	54772
30	exp Autism Spectrum Disorders/	30272
31	autism spectrum disorder.ti,ab.	15479

32	intellectual disabilit*.ti,ab.	16579
33	learning disabilit*.ti,ab.	7720
34	exp Chronic Illness/ or exp Long Term Care/	287878
35	long term condition*.ti,ab.	1772
36	chronic illness.ti,ab.	11294
37	exp substance-related disorders/	279258
38	mental* ill*.ti,ab.	36605
39	drug user.ti,ab.	1696
40	homeless.ti,ab.	8108
41	clients/	20927
42	service user*.ti,ab.	5079
43	adult.ti,ab.	742421
	**Type of Care**	
44	exp Social Services/	17635
45	social care.ti,ab.	5952
46	integrated services/	0
47	integrated care.ti,ab.	4202
	**PROs**	
48	(PRO integration or Clinical PRO application* or telePRO or automated PRO algorithm* or screening purpose* or PRO questionnaire* or Patient-reported outcome questionnaire* or PROM or Patient-reported outcome measure* or Patient reported outcome measure* or Patient-reported symptom* or Patient-centred care or Patient self-report* or Self-report health or Self-rated health or Self-reported measure* of health or Health outcome* or Health communication* or Hospital performance evaluation* or Automated telephone survey system* or paper-based survey* or web-based survey* or web-based PRO platform* or web-based system* or PRO collection* or PRO measure* or PRO intervention* or PRO assessment intervention* or PRO data or PRO assessment* or Routine PRO assessment* or Routine PRO collection or Symptom assessment* or Symptom monitoring or Symptom data or Functional status or Electronic PRO assessment* or Electronic PRO system* or ePRO or ePRO* or ePRO system* or PRO system* or Generic PRO system* or PRO-based clinical alert system*).ti,ab.	107511
49	exp Patient Reported Outcome Measures/	6264
50	((((patient adj reported adj outcome adj measure*) or patient) adj reported adj outcome*) or capability or capabilities).ti,ab.	176329
51	((disability or function or functional or functions or subjective or utility or utilities or wellbeing or well being) adj2 (index or indices or instrument or instruments or measure or measures or questionnaire* or profile or profiles or scale or scales or score or scores or status or survey or surveys)).ti,ab.	124804
52	((patient or self or carer or proxy) adj (appraisal* or appraised or report or reported or reporting or rated or rating or based or assessed or assessment*)).ti,ab.	216843
53	self-report/	32909
54	health status/	81773
55	(health index* or health indices or health status or health profile*).ti,ab.	61364
56	exp "Quality of Life"/	196075
57	quality of life.ti,ab.	272438

58	(HR-PRO or HRPRO or HRQL or HRQoL or QL or QoL).ti,ab.	56723
59	(Patient Centred Care or person centred care or person centred coordinated care or p3c or person centred outcome*).ti,ab. or person reported outcome*.mp. or individualised patient reported outcome measur*.ti,ab. or iprom.ti,ab.	2666
60	patient centered care.ti,ab.	3685
61	person centered care.ti,ab.	763
62	(Patient Centered Care or person centered care or person centered coordinated care or p3c or person centred outcome*).ti,ab. or person reported outcome*.mp. or individualised patient reported outcome measur*.ti,ab. or iprom.ti,ab.	4526
	**Implementation**	
63	((implementation or implementation science).ti,ab. or implementation.mp.) adj3 science.ti,ab. [mp=title, abstract, original title, name of substance word, subject heading word, floating sub-heading word, keyword heading word, organism supplementary concept word, protocol supplementary concept word, rare disease supplementary concept word, unique identifier, synonyms]	1777
64	"barriers and facilitators".ti,ab.	6024
65	1 or 2 or 3 or 4 or 5 or 6 or 7 or 8 or 9 or 10 or 11 or 12 or 13 or 14 or 15 or 16 or 17 or 18	233513
66	19 or 20 or 21 or 22 or 23 or 24 or 25 or 26 or 27 or 28 or 29 or 30 or 31 or 32 or 34 or 35 or 36 or 41 or 42 or 43	1921113
67	44 or 45 or 46 or 47	26784
68	48 or 49 or 50 or 51 or 52 or 53 or 54 or 55 or 56 or 57 or 58 or 59 or 60 or 61 or 62	918146
69	63 or 64	7740
70	65 and 66 and 68	8191
71	65 and 66 and 68 and 69	27
72	65 and 66 and 67 and 68 and 69	0
73	65 and 66 and 67 and 68	188
74	65 or 66 or 67	2133681
75	68 and 74	184887
76	69 and 70 and 74	27
77	limit 73 to yr="2010 - Current"	133
