# Supplementary material for: Patient-reported outcomes in integrated health and social care: A scoping review
Source: JRSM Open. 2024 Mar 24;15(3):20542704241232866. doi: 10.1177/20542704241232866 (PMC10962043; doi:10.1177/20542704241232866)
Supplement: sj-pdf-2-shr-10.1177_20542704241232866 - Supplemental material for Patient-reported outcomes in integrated health and social care: A scoping review [file sj-pdf-2-shr-10.1177_20542704241232866.pdf]

Supplementary Appendix 2: Summary of the included articles – study, population and setting characteristics

Lead Author	Year of publication	Country	Study Design	N	Population	Study Setting
Abbott[1]	2012	United States	Cohort study	216	Older adults (>60 years) Receiving long-term services and supports	Long-term care (including nursing homes, assisted living facilities, and home/community-based services)
Apostolo[2]	2014	Portugal	Randomized Control Trial	56	Adults who are nursing home residents	Nursing homes
Astell[3]	2018	The Netherlands Greece Slovenia Spain Sweden UK	Pilot study	2900	Adults living with cognitive impairment (1163) and other stakeholders (informal carers (n = 140), healthcare professionals (n=10), stakeholders (n = 26) including health & Social care providers, housing, the charitable sector, community services, long-term care).	Various (outpatient clinics, assisted living facilities, long-term care, nursing homes)
Ayalon[4]	2016	Israel	Cohort study	223 complete triads	Caregiving triads composed of an older adult, a family member, and home care worker	Community-dwelling
Ayalon[5]	2019	Isreal	Cross sectional study	245	Older adults from two different long term care settings (day centres and	Two settings – Adult Day Care Centres (ADCC) and Continuing Care Retirement Centres (CRCC)
Bangerter[6]	2016	United States	Qualitative research	337	Nursing home residents	Nursing home
Barczak[7]	2016	Poland	Cohort study	226	Adults (>55 years) living in a family environment or social care centres	Family homes and social care centres

Beresford[8]	2019	UK	Cohort study	64 at T2	Community-dwelling adults referred to social care reablement services	Reablement services in England
Bernardes[9]	2017	Portugal	Validation study	165	Adults with chronic MSK pain	Day care centres
Boniface[10]	2013	UK	Systematic review	N/A	N/A	N/A
Borowiak[11]	2015	Poland	Cohort Study	2627	Older adults (>65 years) living in rural and urban and institutional environments	Urban, rural (community-dwelling) and institutional (nursing home) environments
Bousquet[12]	2019	France	Longitudinal, observational pilot study	138	Elderly people (>65 years) living at home and no more than moderately impaired receiving home care	Community-dwelling
Bower[13]	2018	UK	Mixed methods	Multiple studies (see full text)	Older people (>65 years) health, and social care professionals	Large scale integrated care project
Breysse[14]	2022	United States	Randomised controlled trial	153	Older adults >65 years, with an household income ≤80% of area median income (AMI); did not reside in a direct service medical care facility; had difficulties with ≥1 ADL or ≥2 IADLs; were cognitively intact; were able to stand with or without assistance; had not been hospitalized overnight >4 times in previous year; were not receiving in-home services, outpatient PT services for balance or muscle	Community-dwelling

					strengthening, or active cancer treatment; and were comfortable speaking English	
Brooker[15]	2011	UK	Randomised controlled trial	193	People with dementia and other mental health problems living in extra care housing schemes	Extra-care housing schemes
Burack[16]	2012	United States	Cross sectional study	62	Elderly adults who were nursing home residents	Nursing homes (within a long term care system)
Burton[17]	2022	UK	Personal view /opinion article	N/A	Older adult care home residents	Care home
Callaghan[18]	2014	UK	Cross sectional study	618	Older adults living in care homes, extra care housing, or receiving care at home	Extra care housing, care home, community-dwelling receiving care in own home
Callaghan[19]	2017	UK	Non-randomised experimental study	150	Older adults using Shared Lives support	Shared Lives support (residential, day support, respite, outreach/kinship.)
Cardona[20]	2017	Australia	Psychometric study	224	Adults who received consumer-directed care packages at home in Australia	Not reported
Cardona[21]	2018	Australia	Narrative review	N/A	Aged care population in Australia	Community aged care programmes in Australia
Chao[22]	2021	Taiwan	Cross sectional study	158	Psychiatric nursing home residents	-

Chatterjee[23]	2019	India	Psychometric study	267	Older adults (60+ years)	Various (outpatient dept, care home, community-dwelling)
Chau[24]	2018	Australia	Cross sectional study	228	Adults (aged 65+ years) and residents of long-term care facilities	Residential long term care facilities, dementia-specific facilities were excluded
Claes[25]	2012	Belgium	Psychometric study	Study 1 - 161	People with an intellectual disability	Not specified; participants are service users supported by The Arduin Foundation, The Netherlands
Cochrane[26]	2016	Ireland	Systematic review	811	Community-dwelling older adults (Aged > 65 years) who required assistance with ADLs due to poor mental and/or physical health	Community-dwelling
Conrad[27]	2011	United States	Psychometric study	226	Older adults substantiated for at least one type of elder mistreatment	Protective-service agencies
Cooke[28]	2016	Australia	Pilot study (using before and after (pre/post-test) design)	25	Adults with severe disability (complex care needs) living in residential homes	Residential home
Creighton[29]	2019	Australia	Cross sectional study	178	Adults (>65 years) living in residential aged care facilities (i.e., nursing homes)	Residential aged care facilities

daSilva[30]	2020	Portugal	Non-randomised experimental study	100	Adults >65 years	Care home and Community-dwelling
deHeer-Wunderink[31]	2012	The Netherlands	Cross sectional study	Care coordinators = 119 service users = 534 (N = 653)	Care coordinators and service users supported housing services	Community housing programmes
deVet[32]	2017	The Netherlands	Randomised controlled trial	183	Adults moving from shelters to supported or independent housing	Homeless shelter/Community living
deVries [33]	2021	The Netherlands	Psychometric study	12	Palliative care patients (with a life expectancy of <12 months)	Various (participants' preferred location), participants recruited from 2 care homes, a general hospital and hospice
Dewitte[34]	2018	Belgium	Case control study	88 Resident-staff pairs	Older adults (>65 years) with Alzheimer disease in residential care	7 residential care homes
Dezutter[35]	2020	Belgium	Cross sectional study	327	Adults living in nursing homes (no cognitive, hearing or speech impairment)	Nursing home

Diefenbach[36]	2012	United States	Cross sectional study	66	Older adults (>65 yrs.) who were clients of a home care programme for low-income and functionally disabled elderly persons.	Community-dwelling
Dobrzyn-Matusiak[37]	2014	Poland	Cross sectional study	300	Elderly adults	Long term care institutions, adult day care homes, community-dwelling
Donald[38]	2022	Canada	Qualitative study	6	People with lived experience of homelessness and chronic illness	Homelessness
Dubuc[39]	2011	Canada	Non-randomised experimental study	746	Older adults (75+) living in the community and at risk of functional decline	Community-dwelling
Ellis-Smith[40]	2021	UK	Systematic review	Range 13 - 289,753	Adults aged 65 years and over, living with advanced or life-limiting condition(s)	Services or facilities associated with advanced disease (e.g., receiving palliative care, residing in a care home)
Elston[41]	2019	UK	Economic evaluation	86	Participants were individuals aged 50 years or over with two or more long-term conditions, considered as likely to benefit from a social intervention.	-
Farina[42]	2021	Germany	Qualitative research	35	Professionals with different levels of qualification (secondary and tertiary), care personnel without specific qualification, and family carers of people with dementia. All participants provided care for people with advanced dementia in nursing homes or home care.	Nursing Homes, Own Home Care

Forder[43]	2018	UK	Cross sectional study	622 cases	People aged 40+ years	Data from the Adult Social care Survey 2012/2013
Gerber[44]	2016	Canada	Cohort study	63	Persons who had sustained an ABI within the past 4 years, were receiving publicly funded community services and were able to understand and read basic English were invited to participate in the study.	Community-dwelling, receiving services from publicly funded community care access centre
Gerolimatos[45]	2013	United States	Psychometric study	75	Nursing home residents	Nursing home
Gethin-Jones[46]	2012	UK	Pilot study	40	Older adults (65+ years) with critical and substantial care needs	Home care
Ghesquiere[47]	2018	United States	Qualitative research	16 palliative care providers	Aging service providers (primarily social workers)	2 social services centres in New York state
Glasby[48]	2019	UK	Mixed methods	74	Quantitative stage (PRO collection) = older adults being resettled (from either a care home or day centre identified for closure)	Care home and day centres
Godin[49]	2015	Canada	Psychometric study	1578 (residents = 319)	Staff, residents, family members	Nursing home
Goodwin[50]	2014	UK	Cross sectional study	275 (193 at 4 weeks follow-up)	Adults with advanced cancer receiving palliative care	Hospice
Gordon[51]	2012	United States	Cross sectional study	7235	Homeless adults receiving intensive case management services	homeless adults

Gradinger[52]	2020	UK	Mixed methods cross-case analysis	49	Adults who received Wellbeing Coordination Services (WBC)	Community-dwelling and accessing the WBC services
Grohetal[53]	2013	Germany	Non-randomised experimental study	60	Adults with advanced life-limiting illness receiving SOPC services at home.	Palliative home care service (Community-dwelling)
Guerrero[54]	2021	Czech Republic	Mixed Methods Study	870	- Twenty-seven institutions across the Czech Republic<br>- Clients, staff and management surveyed<br>*When parents' informed consent was needed for child participants to take part in the survey, they were excluded from the study.	"Residential treatment centres, foster homes, child diagnostic institutes, elderly homes, nursing homes, homes with special arrangements and psychiatric hospitals. "
Hajji[55]	2020	Austria	Psychometric study	1000	Representative sample of Austrian adults	General population (community-based)
Hall[56]	2012	United States	Psychometric study	108	Older adults (>65 years) residing in continuing care retirement communities	Continuing care retirement facility (63% independent living; 35.2% assisted living)
Hanratty[57]	2018	UK	Cross sectional study	254 cases + 1016 matched controls	Adults aged >50 years	Care home (cases), community-dwelling (matched controls)

Hansen[58]	2016	United States	Cross sectional study	96	Urban community-dwelling older adults (n = 96) 65 years of age and older with Adult Protective Services-substantiated self-neglect	Community-dwelling
Harding[59]	2012	Multi-country study (South Africa and Uganda)	Cross sectional study	230	Adult patients (at least 18yrs.) with a confirmed HIV diagnosis known to the patient, with sufficient physical and cognitive ability to participate in interviews.	Five facilities serving urban/per-urban population offering a range of integrated palliative care services
Harding[60]	2014	Uganda, Kenya	Cross sectional study	210	Adults (>18 years) with advanced cancer in Africa	Hospice
Herce[61]	2014	Malawi	Cohort study	Patient participants: 63	Patients with chronic conditions enrolled in the Neno Palliative Care Program	NPCP in a variety of settings including outpatient clinic and home visits
Hernandez[62]	2020	United States	Cross sectional study	567	Adults experiencing homelessness	Homeless shelters
Hirani[63]	2014	UK	Randomised controlled trial	1,189	Adults (>18 years) receiving local authority Social care	Not reported (participants recruited from LA Social care databases)
Huang [64]	2022	Australia	Cross-sectional study	474	General population of adults 20+ years	Community dwelling
Huntley[65]	2015	United States	Cohort study	60	Adults (>18 years) attending a breakfast program	Weekly breakfast programme (voluntary sector)

Jarrott[66]	2019	United States	Delphi panel	12	ADS researchers and practitioners	Remote/survey-based
Jeste[67]	2019	United States	Cross sectional study	223 (Senior housing = 104; SAGE = 119)	Senior housing residents who were English-speaking adults, 65 years who could complete study assessments, and with no known diagnosis of dementia or any other disabling illness and comparison group identified from SAGE study	Continuing care senior housing community
Kaambwa[68]	2015	Australia	Psychometric study	87	Community-swelling older adults (>65years), cognitively intact, receiving aged care services	Community-dwelling and receiving aged care services
Kehusmaa[69]	2012	Finland	Economic evaluation	732	Adults > 65 years with progressively decreasing functional ability and risk of institutionalization within 2 years	Unclear, multiple settings
Kleisiaris[70]	2019	Greece	Cross sectional study	120	Older adults (>65 years) enrolled in a home care program	Community-dwelling receiving home care services
Lau[71]	2021	Singapore	Systematic review	781 patients with advanced cancer	Patients with advanced cancer	-
Lee[72]	2013	United States	Cross sectional study	610	Adults aged 60 and above entering public community long term care (derived from the baseline survey of a longitudinal study).	Community-dwelling (receiving continuing long term care including nursing, case management, personal)
Lee[73]	2016	Korea	Cross sectional study	178 patient-caregiver pairs	Palliative care patients with a terminal cancer diagnosis	Palliative care units in South Korean hospitals

Leung[74]	2019	UK	Cross sectional study	204	Older adults (>55 years) living in sheltered housing	Sheltered housing
Liao[75]	2022	Taiwan	Randomised controlled trial	143	Community-dwelling older adults with a medical diagnosis of depressive disorder or depressive mood (>55years old) .	Community-dwelling
Liimatta[76]	2020	Finland	Randomised controlled trial	422	Independent, home-dwelling older adults (>75 years)	Community-dwelling
Liu[77]	2018	China	Cross sectional study	1452	Elderly Chinese nursing home residents (60 - 99 years)	Nursing homes
Low[78]	2015	Australia	Non-randomised experimental study	189	Adults who were clients of an Australian aged care program (home care)	Community-dwelling receiving home care
Low[79]	2018	Australia	Pre-post mixed methods design	Residents = 69<br>Staff = 85	Residents and staff of aged care facilities that participated in the LifeFul reablement-focused culture change program	Aged care facilities
Lu[80]	2021	Hong Kong	Cross sectional study	2707	Adults aged 60 years and over and have mild or above depressive symptoms	Community-dwelling
Malley[81]	2012	UK	Psychometric study	301	Older people receiving home care	Community-dwelling
Matos[82]	2013	Portugal	Psychometric study	151	Older adults	Day care centre (33.1%), nursing home (36.4%) or senior university (30.5%).

McGilton[83]	2021	Canada	Other: Scoping review	-	TCP patients were typically older women with multiple chronic conditions and some cognitive impairment, functionally dependent, and living alone.	Long-term care homes, acute care hospitals, SNFs, community and rural hospitals.
Meeks[84]	2016	United States	Cross sectional study	26	Long term care patients	Long term care facilities
Milberg[85]	2014	Sweden	Cross sectional study	168	Adults (18+) with a diagnosis of non-curable disease and an expected short survival admitted to a palliative care unit.	palliative home care unit
Mitolo[86]	2017	Italy	Randomised controlled trial	30	Older adults (70-90 years) who were residential care home residents	residential care home
Munford[87]	2020	UK	Cohort study	2449	Older adults (65+) with long term conditions	Not reported (population-level study)
Murphy[88]	2015	Ireland	Cross sectional study	3507	Community-dwelling adults aged 65+ years	Community-dwelling

Nakamura-Thomas[89]	2019	Japan	Psychometric study	1102	Japanese community-dwelling people in receipt of Social care services	Community-dwelling and receipt of Social care services
Naylor[90]	2016	United States	Cohort study	470	Older adults (60+ years) without severe cognitive impairment who were first time recipients of LTSS and, at the time of enrolment, receiving services from one of the following common providers: home and community-based services (H&CBS), assisted living facilities (ALFs), or nursing homes (NHs).	Assisted living (33.19%); nursing home (33.62%); home and community-based (33.19%)
Negron-Blanco[91]	2016	Spain	Cross sectional study	1622	Adults aged 50+ years	Community-dwelling
Netten[92]	2012	UK	Psychometric/ health economic study	1130 (2 phases)	Users of adult social care	Multiple social care settings
Netten[93]	2012	UK	Cohort study	366	Residents of care homes for older people	Care homes in England (n = 83)
Neves[94]	2018	Canada	Case series	5 dyads = residents + relative	Chinese Canadian frail institutionalized oldest-old and five relatives (pseudonyms used throughout)	Long term care home

Nguyen[95]	2021	Finland	Psychometric study	493 self-completed; 334 proxy respondents	Randomly chosen regular home care user aged 55 years or above.	Home care
Nicholson[96]	2019	Ireland	Non-randomised experimental study	Participants were people with mild/moderate intellectual disabilities (n=82) and their keyworkers (n=81).	Adults with mild-moderate intellectual disabilities receiving respite care	Respite care settings (n = 3)
Nikmat[97]	2015	Malaysia	Non-randomised experimental study	219	Older adults (60-89 years) with cognitive impairment	Nursing homes and home care
O'Riley[98]	2014	United States	Cross sectional study	377	Community-dwelling older adults (60+ years) who sought social services through one Monroe County ASN agency between September 2005 and August 2007	Community-dwelling
Oliveira[99]	2019	Portugal	Cross sectional study	115	Older adults (65-98 years) enrolled in the home care support and adult day care system in the county of Coimbra, Portugal	Two settings (adult day care = 85 (73.9%) and home care = 30 (26.1%)
Ornstein[100]	2013	United States	Cross sectional study	140	Homebound adults (>18 years) newly enrolled in the HBPC programme	Community-dwelling

Ozen[101]	2019	Turkey	Cross sectional study	126	Elderly nursing home residents	Nursing home
Padmakar[102]	2020	India	Other: Mixed-methods evaluation	11	Mental health service users who transitioned from a psychiatric in-patient unit to independent living facilities	Supported housing for people with MH problems who are transitioning from being inpatients
Park[103]	2014	Korea	Cross sectional study	697	Adults aged 50+ years using home care services	Community-dwelling
Park[104]	2014	Korea	Randomised controlled trial	43	Older adults 65+ years with multi-morbidity (2 or more diagnoses of chronic diseases within 1 year prior to the study)	Nursing home
Park[105]	2019	Korea	Psychometric study	216	Adults with dementia or mild cognitive impairment resident in nursing homes	Nursing homes
Parker[106]	2017	UK	Secondary analysis of trial data	556 dyads (residents and care staff); residents were adults 65+ years	Care home residents	78 care homes in 2 regions in England
Pesut[107]	2017	Canada	Pilot study	25	Older adults (55+) living with chronic illness (and family members)	Community-dwelling, rural setting
Phillipson[108]	2022	Australia	Qualitative study	63	Older adults who were recipients of home care	Community dwelling

Potter[109]	2017	UK	Psychometric study	1211	Adults with long term conditions	Postal survey with a diverse group of health and social care users
Pramesona[110]	2018	Indonesia	Cross sectional study	181	Older adults 60+ years and nursing home residents	Nursing homes
Rand[111]	2017	UK	Cross sectional study	13, 642	Adults with intellectual disabilities who participated in the Adult Social care Survey in England	Postalsurvey
Rand[112]	2017	UK	Psychometric study	770	Adult (+18yrs) who were users of Social care services with primary reason for support being physical disability or learning disability or mental health condition or sensory impairment	Community-dwelling, in receipt of community-based social services
Rand[113]	2020	UK	Psychometric study	264	Adults (+18yrs) with IDD who were able to consent, complete the questionnaire alone or with support, and were users of social care services	Various
Rand[114]	2021	UK	Cohort study	313	Carers in England (+18yrs), providing help or support to someone living with dementia, who uses community-based Social care, and does not live in residential or nursing care and is unable to self-complete a structured questionnaire, even with help.	Community-dwelling

Raven[115]	2018	United States	Cohort study	300	Homeless adult aged 50+ years	Homeless encampments, overnight homeless shelters, free meal programs
Reinhardt[116]	2014	United States	Phase II pilot study	37	Residents of LTC facilities (recruited from major geriatric nursing home) with intact cognitive function, sufficient hearing, English speaking and able to communicate verbally	Nursing home
Richardson[117]	2012	United States	Cohort study	378	Community-dwelling older adults (60+ years)	Community-dwelling accessing Eldersource services
Rikkert[118]	2018	The Netherlands	Text and opinion	Not reported / N/A	Geriatric people	Not reported / N/A
Saunders[119]	2021	Canada	Mixed Methods	39	Adult inpatients and their caregivers receiving palliative care consultation and transitioned to home-based palliative care.	In-hospital
Sheffield[120]	2013	United States	Randomised controlled trial	71	Adults (65+ years) receiving agency service who were community-dwelling seniors who had significant impairments in ADLs.	Community-dwelling
Shippee[121]	2015	United States	Cross sectional study	10,969	Care home residents	Care home

Siegert[122]	2014	Sub-Saharan Africa (Kenya, South Africa, Uganda)	Psychometric study	461	Adults (+18yrs) with confirmed HIV and/or cancer diagnosis under palliative care	Various palliative care settings
Siette[123]	2018	Australia	Qualitative research	12	Aged care staff	Aged care organisation offering a range of care settings including residential, home and outreach services.
Siette[124]	2020	Australia	Cross sectional study	175	Older adults receiving aged care services in Australia	Home and community-based care
Simning[125]	2019	United States	Cohort study	112	Patients (65+ years) at a skilled nursing facility with a plan to be discharged home	Skilled nursing facility rehabilitation units
Sirey[126]	2013	United States	Cohort study	137	Homebound older adults 60+ years who are eligible for home-delivered meals and who endorse depressive symptoms upon screening	Community-dwelling receiving home meals service
Siverova[127]	2018	Czech Republic	Non-randomised experimental study	116	Adults (>60 years) who were long term care clients presenting with cognitive impairment	Care home (long term care facility)

Smelson[128]	2016	United States	Pilot feasibility study	107	People with co-occurring disorders and experiencing homelessness	Supportive housing
Smith[129]	2018	UK	Other: Observational study, secondary data analysis	72	Older adults who were residents of a care home (nursing or residential) in the UK	Care homes
Smith[130]	2021	United States	Observational study	10915	Patients Seen by Specialty Palliative Care Teams at Home Versus in Clinic, 18 years and older.	Clinics and at Home
Spoorenberg[131]	2018	The Netherlands	Randomised controlled trial	1456	Older adults (+75 years)	Community-dwelling
Spoorenberg[132]	2019	The Netherlands	quasi-experimental (one group pre/post-test design)	136	Older adults (>75 years) receiving the person-centred, integrated care service Embrace who were classified as frail and as having complex care needs	Community-dwelling
Stergiopoulos[133]	2015	Canada	Randomised controlled trial	378	Ethnically diverse homeless adults (18+ years) with mental health disorder	Housing first

Szanton[134]	2019	United States	Randomised controlled trial	330	Adults (65+ years), cognitively intact with reported difficulty in at least 1 ADL, on low-income.	Community-dwelling
Tay[135]	2021	Singapore	Cohort study	359	Deceased, adult (aged >21years) patients with advanced cancer who were enrolled in the home-based palliative care service from January 2016 to December 2018.	Home-based
Taylor[136]	2016	United States	Pilot study	47 residents, social service coordinator (n = 1) for carer-reported assessment	Residents living in a senior housing apartment complex	Senior housing communities
Tazopoulou[137]	2016	France	Non-randomised experimental study	20	Institutionalised adults with cerebral anoxia in residential care facilities	Residential care
Toles[138]	2023	United States	Randomised controlled trial	327 dyads	Skilled nursing facility patients and their caregivers who transition to home	Integrated care
Tosangwarn [139]	2018	Thailand	Cross sectional study	128	Older residents (60+ years) of care homes	Care home
Towers[140]	2015	UK	Qualitative research	Consultation = 28 stakeholders (+ 17 people	Mixed - The workshop attendees (N= 28) included care home providers, local authority staff, representatives from membership body for the voluntary care	N/A

				with caring responsibilities; Feasibility study = 5 quality monitoring officers in two care homes	sector, Skills for Care, Health Watch, NICE, SCIE and professionals involved in education and training in the sector (e.g., around end-of-life care). We also interviewed representatives from a membership body for the nursing home sector (N= 2), a local authority unable to attend the workshop (N= 1) and the health and social care regulator (CQC) (N= 2). Professional stakeholders came from several regions of England including the Southeast, London, the Midlands, Northeast, and the North West. Lay stakeholders (relatives and carers of older people living in care homes, relatives, and carers of people with experience of Social care services and support, adults aged between 45 and 75 who may have to help arrange residential care for an older relative in the future but yet have no experience of a care home).	
Towers[141]	2016	UK	Mixed-methods feasibility study of feedback intervention for care homes	Residents = 58	Care home residents and staff	Nursing and residential care homes (n = 4)
Travers[142]	2011	Australia	Non-randomised experimental study	113	Older adults (60+ years) - Community-dwelling and residents of residential care facilities	Community-dwelling and residential care facilities
Turnpenny[143]	2018	UK	Qualitative research	54	Adults with intellectual disabilities and/or autism	Not applicable
Uittenbroek[144]	2021	The Netherlands	Randomised controlled trial	1456	Older adults (75+ years) identified from GP practices who were not already enrolled in long term care or other similar care service.	Integrated primary care (GP practices)

Valdovinos[145]	2021	United States	Scoping review	Range 10-3521	Secondary data analysis of primary studies	Not applicable as review is focussed on patient portal tools.
vanLeeuwen[146]	2014	The Netherlands	Cross sectional study	29,935	English adult social care service users (65+ years) living at home	Community-dwelling
vanLeeuwen[147]	2015	The Netherlands	Psychometric study	190	The study population consisted of a sample of participants of the ‚Äúfrail older Adults: Care in Transition‚Äù(ACT) study in The Netherlands.	Community-dwelling
Waldrop[148]	2015	United States	Cohort study	42	Older adults with cancer	Hospice/Non-hospice
Walshe[149]	2016	UK	Randomised controlled trial	196	Participants in the trial include people anticipated to be in their last year of life and their self-identified in-formal carer.	Community-dwelling, participants were service users of end-of life home care services.
Watkins[150]	2012	United States	Other: single group pre/post design	292	Adults (>65 years) enrolled in the Hospital to Home programme	Reablement/transitional care at home
Whitehead[151]	2018	UK	Randomised controlled trial	60	Adults (>65 years) referred by a Social care occupational therapy team member for the provision of an accessible showering facility. Exclusion criteria were; referral for an accessible showering facility plus one or more other adaptations (e.g. hoist, ramp, lift), and priority, referrals (those fast-tracked based on clinical assessment)	Community-dwelling

Wilberforce[152]	2018	UK	Psychometric study	596	Home-dwelling service users on the active caseloads of integrated community mental health and social care services for older people.	Community-dwelling
Wilner[153]	2013	UK	Randomised controlled trial	179	People with intellectual disabilities identified as having problems with anger control	Day service for people with ID
Wong[154]	2022	Hong Kong	Randomised controlled trial	221	Participants included older adults aged at least 60 years who were livingwithin the service area, used a smartphone, and had at least 1 of the following problems: chronic pain, hypertension, or diabetes.	Community centres
Woolham[155]	2017	UK	Cross sectional study	634	Older people (75+) in receipt of a personal budget	Various
Xiang[156]	2020	China	Cross sectional study	192	Resident from nursing homes. Most participants were 75 years or older.	Care home (nursing)
Yang[157]	2017	UK	Economic evaluation	9 local authorities in England	Local authority level data	Adult social care services with a focus on care homes
Yao[158]	2019	Taiwan	Non-randomised experimental study	55	Older adults living in nursing homes	Nursing home

Ysseldyk[159]	2013	Canada	Cross sectional study	Study 1 only n = 42	Old adults living a care homes (either independent living or assisted care)	Care home

References

1. Abbott KM, Bettger J, Hanlon A, et al. Factors associated with health discussion network size and composition among elderly recipients of long-term services and supports. *Health Commun* 2012; 27: 784–793.

2. Apostolo JLA, Cardoso DFB, Rosa AI, et al. The Effect of Cognitive Stimulation on Nursing Home Elders: A Randomized Controlled Trial. *J Nurs Scholarsh* 2014; 46: 157–166.

3. Astell AJ, Gradisek A, Bizjak J, et al. INLIFE - Independent Living Support Functions for the Elderly: Technology and Pilot Overview. In: Chatzigiannakis I, Tobe Y, Novais P, et al. (eds) *Intelligent Environments 2018*. Amsterdam: Ios Press, pp. 526–535.

4. Ayalon L, Roziner I. Satisfaction with the relationship from the perspectives of family caregivers, older adults and their home care workers. *Aging Ment Health* 2016; 20: 56–64.

5. Ayalon L. Subjective social status as a predictor of loneliness: The moderating effect of the type of long-term care setting. *Res Aging* 2019; 41: 915–935.

6. Bangerter LR, Van Haitsma K, Heid AR, et al. 'Make Me Feel at Ease and at Home': Differential Care Preferences of Nursing Home Residents. *Gerontologist* 2016; 56: 702–713.

7. Barczak K, Szmidt M, Buczkowska-Radlinska J. The functionality of masticatory apparatus and the sense of depression in patients over 55 years of age living in a family environment and social care centres. *Funkcjonalnosc narzadu zucia a poczucie depresji u pacjentow po 55 roku zycia zamieszkalych w srodowisku rodzinnym i w domach pomocy spolecznej* 2016; 50: 1027–1038.

8. Beresford B, Mayhew E, Duarte A, et al. Outcomes of reablement and their measurement: Findings from an evaluation of English reablement services. *Health soc care community* 2019; 27: 1438–1450.

9. Bernardes SF, Matos M, Goubert L. Older adults' preferences for formal social support of autonomy and dependence in pain: development and validation of a scale. *Eur J Ageing* 2017; 14: 257–268.

10. Boniface G, Mason M, Macintyre J, et al. The effectiveness of local authority social services' occupational therapy for older people in Great Britain: A critical literature review. *Br J Occup Ther* 2013; 76: 538–547.

11. Borowiak E, Kostka J, Kostka T. Comparative analysis of the expected demands for nursing care services among older people from urban, rural, and institutional environments. *Clin interv aging* 2015; 10: 405–12.

12. Bousquet J, Meissonnier M, Michalet V, et al. A novel approach to integrated care using mobile technology within home services. The ADMR pilot study. *Maturitas* 2019; 129: 1–5.

13. Bower P, et al. Improving care for older people with long-term conditions and social care needs in Salford: the CLASSIC mixed-methods study, including RCT. *Health Services and Delivery Research*; 6. Epub ahead of print 2018. DOI: 10.3310/hsdr06310.

14. Breyesse J, Dixon S, Wilson J, et al. Aging Gracefully in Place: An Evaluation of the Capability of the CAPABLE(C) Approach. *J Appl Gerontol* 2022; 41: 718–728.

15. Brooker DJ, et al. The Enriched Opportunities Programme for people with dementia: a cluster-randomised controlled trial in 10 extra care housing schemes. *Aging Ment Health* 2011; 15: 1008–1017.

16. Burack OR, Weiner AS, Reinhardt JP, et al. What matters most to nursing home elders: Quality of life in the nursing home. *J Am Med Dir Assoc* 2012; 13: 48–53.

17. Burton JK, Wolters AT, Towers AM, et al. Developing a minimum data set for older adult care homes in the UK: exploring the concept and defining early core principles. *Lancet Healthy Longev* 2022; 3: e186–e193.

18. Callaghan L, Towers A-M. Feeling in control: comparing older people's experiences in different care settings. *Aging soc* 2014; 34: 1427–1451.

19. Callaghan L, Brookes N, Palmer S. Older people receiving family-based support in the community: a survey of quality of life among users of 'Shared Lives' in England. *Health soc care community* 2017; 25: 1655–1666.

20. Cardona B, Fine M, Stebbing A, et al. Measuring consumer outcomes: Development and testing of the Australian Community Care Outcomes Measure. *Australas J Aging* 2017; 36: 69–71.

21. Cardona B. Measuring outcomes of community aged care programs: Challenges, opportunities and the Australian Community Outcomes Measurement ACCOM tool. *Health Qual Life Outcomes* 2018; 16: 104.

22. Chao PY, Hsieh WL, Yeh ST, et al. Factors Associated with Personal Recovery Among Psychiatric Nursing Home Residents. *Psychiatric Ment Health Nurs* 2022; 27: 1–9.

23. Chatterjee P, Rebok GW, Dwivedi SN, et al. Development of Integrated Care Tool - BRIEF for Screening the Unmet Psychosociomedical Needs of Older Indians. *Indian J Public Health* 2019; 63: 51-+.

24. Chau R, Kissane DW, Davison TE. Risk Factors for Depression in Long-term Care: A Prospective Observational Cohort Study. *Clin Gerontol*; 14.

25. Claes C, et al. Relationship between self-report and proxy ratings on assessed personal quality of life-related outcomes. *J Policy Pract Intellect Disabil* 2012; 9: 159–165.

26. Cochrane A, Furlong M, McGilloway S, et al. Time-limited home-care reablement services for maintaining and improving the functional independence of older adults. *The Cochrane database of systematic reviews* 2016; 10: CD010825.

27. Conrad KJ, et al. Self-report measure of psychological abuse of older adults. *Gerontologist* 2011; 51: 354–366.

28. Cooke M, Emery H, Brimelow R, et al. The impact of therapeutic massage on adult residents living with complex and high level disabilities: A brief report. *Disabil Health J* 2016; 9: 730–734.

29. Creighton AS, Davison TE, Kissane DW. The Factors Associated With Anxiety Symptom Severity in Older Adults Living in Nursing Homes and Other Residential Aged Care Facilities. *J Aging Health* 2019; 31: 1235–1258.

30. da Silva RM, Afonso P, Fonseca M, et al. Comparing sleep quality in institutionalized and non-institutionalized elderly individuals. *Aging Ment Health* 2020; 24: 1452–1458.

31. de Heer-Wunderink C, Visser E, Caro-Nienhuis A, et al. Supported housing and supported independent living in the Netherlands, with a comparison with England. *Community Ment Health J* 2012; 48: 321–327.

32. de Vet R, Beijersbergen MD, Jonker IE, et al. Critical Time Intervention for Homeless People Making the Transition to Community Living: A Randomized Controlled Trial. *Am J Community Psychol* 2017; 60: 175–186.

33. de Vries SRNMs, Lormans TMA, de Graaf ERNP, et al. The Content Validity of the Items Related to the Social and Spiritual Dimensions of the Utrecht Symptom Diary-4 Dimensional From a Patient's Perspective: A Qualitative Study. *J Pain Symptom Manage* 2021; 61: 287–294.

34. Dewitte L, Vandenbulcke M, Dezutter J. Cognitive functioning and quality of life: Diverging views of older adults with Alzheimer and professional care staff. *International J Geriatr Psychiatry* 2018; 33: 1074–1081.

35. Dezutter J, Toussaint L, Dewitte L. Finding a Balance Between Integrity and Despair: A Challenging Task for Older Adults in Residential Care. *J Adult Dev* 2020; 27: 147–156.

36. Diefenbach GJ, Tolin DF, Gilliam CM. Impairments in life quality among clients in geriatric home care: associations with depressive and anxiety symptoms. *Int J Geriatr Psychiatry* 2012; 27: 828–835.

37. Dobrzyn-Matusiak D, Marcisz C, Bak E, et al. Physical and mental health aspects of elderly in social care in Poland. *Clin Interv Aging* 2014; 9: 1793–802.

38. Donald EE, Whitlock K, Dansereau T, et al. A codevelopment process to advance methods for the use of patient-reported outcome measures and patient-reported experience measures with people who are homeless and experience chronic illness. *Health Expect* 2022; 25: 2264–2274.

39. Dubuc N, Dubois MF, Raiche M, et al. Meeting the home-care needs of disabled older persons living in the community: does integrated services delivery make a difference? *BMC Geriatr* 2011; 11: 13.

40. Ellis-Smith C, Tunnard I, Dawkins M, et al. Managing clinical uncertainty in older people towards the end of life: a systematic review of person-centred tools. *BMC Palliat Care* 2021; 20: 168.

41. Elston J, Gradinger F, Asthana S, et al. Does a social prescribing ‘holistic’ link-worker for older people with complex, multimorbidity improve well-being and frailty and reduce health and social care use and costs? A 12-month before-and-after evaluation. *Prim Health Care Res Dev* 2019; 20: 10.

42. Farina H, Hock H, Ellis-Smith C, et al. Culture in the spotlight—cultural adaptation and content validity of the integrated palliative care outcome scale for dementia: A cognitive interview study. *Palliat Med* 2021; 35: 962–971.

43. Forder J, Vadean F, Rand S, et al. The impact of long-term care on quality of life. *Health Econ* 2018; 27: E43–E58.

44. Gerber GJ, Gargaro J, McMackin S. Community integration and health-related quality-of-life following acquired brain injury for persons living at home. *Brain Inj* 2016; 30: 1552–1560.

45. Gerolimatos LA, Gregg JJ, Edelstein BA. Assessment of anxiety in long-term care: examination of the Geriatric Anxiety Inventory (GAI) and its short form. *Int Psychogeriatr* 2013; 25: 1533–1542.

46. Gethin-Jones S. Outcomes and well-being part 1: a comparative longitudinal study of two models of homecare delivery and their impact upon the older person self-reported subjective well-being. *Work Older People* 2012; 16: 22–30.

47. Ghesquiere A, Gardner DS, McAfee C, et al. Development of a community-based palliative care screening tool for underserved older adults with chronic illnesses. *Am J Hosp Palliat Care* 2018; 35: 929–937.

48. Glasby J, Allen K, Robinson S. “A game of two halves?” Understanding the process and outcomes of English care home closures: qualitative and quantitative perspectives. *Soc Policy Adm* 2019; 53: 78–98.

49. Godin K, Stapleton J, Kirkpatrick SI, et al. Applying systematic review search methods to the grey literature: a case study examining guidelines for school-based breakfast programs in Canada. *Syst Rev* 2015; 4: 138.

50. Goodwin L, Price A, Lee W, et al. I’ve had a good life, what’s left is a bonus: Factor analysis of the Mental Adjustment to Cancer Scale in a palliative care population. *Palliat Med* 2014; 28: 243–255.

51. Gordon RJ, Rosenheck RA, Zweig RA, et al. Health and Social Adjustment of Homeless Older Adults With a Mental Illness. *Psychiatr Serv* 2012; 63: 561–568.

52. Gradinger F, Elston J, Asthana S, et al. Integrating the voluntary sector in personalised care: mixed methods study of the outcomes from wellbeing co-ordination for adults with complex needs: Managing Community Care. *J Integr Care* 2020; 28: 405–418.

53. Grohetal G, Vyhnalek B, Feddersen B, et al. Effectiveness of a specialized outpatient palliative care service as experienced by patients and caregivers. *J Palliat Med* 2013; 16: 848–856.

54. Guerrero Z, Aliev A-A, Kondrátová L, et al. Mental Health and Quality & Safety of Care in Czech Residential Institutions during the COVID-19 Pandemic: A Mixed-Methods Study. *Psychiatr Q* 2021; 92: 1393–1411.

55. Hajji A, Trukeschitz B, Malley J, et al. Population-based preference weights for the Adult Social Care Outcomes Toolkit (ASCOT) for service users for Austria: Findings from a best-worst experiment. *Soc Sci Med* 2020; 250: 10.

56. Hall KS, Wojcicki TR, Phillips SM, et al. Validity of the Multidimensional Outcome Expectations for Exercise Scale in Continuing-Care Retirement Communities. *J Aging Phys Act* 2012; 20: 456–468.

57. Hanratty B, Stow D, Moore DC, et al. Loneliness as a risk factor for care home admission in the English Longitudinal Study of Ageing. *Age Ageing* 2018; 47: 896–900.

58. Hansen MC, et al. Correlates of depression in self-neglecting older adults: a cross-sectional study examining the role of alcohol abuse and pain in increasing vulnerability. *J Elder Abuse Negl* 2016; 28: 41–56.

59. Harding R, Selman L, Agupio G, et al. Intensity and correlates of multidimensional problems in HIV patients receiving integrated palliative care in sub-Saharan Africa. *Sex Transm Infect* 2012; 88: 607–611.

60. Harding R, Powell RA, Namisango E, et al. Palliative care-related self-report problems among cancer patients in East Africa: a two-country study. *Support Care Cancer* 2014; 22: 3185–3192.

61. Herce ME, Elmore SN, Kalanga N, et al. Assessing and Responding to Palliative Care Needs in Rural Sub-Saharan Africa: Results from a Model Intervention and Situation Analysis in Malawi. *PLoS One* 2014; 9: 17.

62. Hernandez DC, Daundasekara SS, Zvolensky MJ, et al. Urban Stress Indirectly Influences Psychological Symptoms through Its Association with Distress Tolerance and Perceived Social Support among Adults Experiencing Homelessness. *Int J Environ Res Public Health* 2020; 17: 15.

63. Hirani SP, Beynon M, Cartwright M, et al. The effect of telecare on the quality of life and psychological well-being of elderly recipients of social care over a 12-month period: the Whole Systems Demonstrator cluster randomised trial. *Age Ageing* 2014; 43: 334–41.

64. Huang Y-L, Yates P, Thorberg FA, et al. Influence of social interactions, professional supports and fear of death on adults' preferences for life-sustaining treatments and palliative care. *Int J Nurs Pract* 2022; 28: e12940.

65. Huntley SS. A comparison of substance abuse severity among homeless and non-homeless adults. *J Hum Behav Soc Environ* 2015; 25: 312–321.

66. Jarrott S, Ogletree AM. Adult Day Services Outcomes: Delphi Review of an Integrated Participant Assessment System. *J Appl Gerontol* 2019; 38: 386–405.

67. Jeste DV, Glorioso D, Lee EE, et al. Study of Independent Living Residents of a Continuing Care Senior Housing Community: Sociodemographic and Clinical Associations of Cognitive, Physical, and Mental Health. *Am J Geriatr Psychiatr* 2019; 27: 895–907.

68. Kaambwa B, Gill L, McCaffrey N, et al. An empirical comparison of the OPQoL-Brief, EQ-5D-3 L and ASCOT in a community dwelling population of older people. *Health Qual Life Outcomes* 2015; 13: 164.

69. Kehusmaa S, Autti-Ramo I, Helenius H, et al. Factors associated with the utilization and costs of health and social services in frail elderly patients. *BMC Health Serv Res* 2012; 12: 204.

70. Kleisiaris CF, Papathanasiou IV, Tsaras K, et al. Factors affecting the health status of elderly people receiving home care. *Arch Hell Med* 2019; 36: 237–244.

71. Lau J, Khoo AM, Ho AH, et al. Psychological resilience among palliative patients with advanced cancer: A systematic review of definitions and associated factors. *Psycho-Oncology* 2021; 30: 1029–1040.

72. Lee MJ, Hasche LK, Choi S, et al. Comparison of major depressive disorder and subthreshold depression among older adults in community long-term care. *Aging Ment Health* 2013; 17: 461–469.

73. Lee YJ, Kim JE, Choi YS, et al. Quality of life discordance between terminal cancer patients and family caregivers: a multicenter study. *Supportive Care Cancer* 2016; 24: 2853–2860.

74. Leung P, et al. Emotional distress mediates the relationship between cognitive failures, dysfunctional coping, and life satisfaction in older people living in sheltered housing: a structural equation modelling approach. *Int J Geriatr Psychiatry* 2019; 34: 179–185.

75. Liao SJ, Chao SM, Fang YW, et al. The Effectiveness of the Integrated Care Model among Community-Dwelling Older Adults with Depression: A Quasi-Randomized Controlled Trial. *IJERPH* 2022; 19: 3306.

76. Liimatta HA, Lampela P, Kautiainen H, et al. The Effects of Preventive Home Visits on Older People's Use of Health Care and Social Services and Related Costs. *The journals of gerontology Series A, Biological sciences and medical sciences* 2020; 75: 1586–1593.

77. Liu YB, Xue LL, Xue HP, et al. Health literacy, self-care agency, health status and social support among elderly Chinese nursing home residents. *Health Educ J* 2018; 77: 303–311.

78. Low LF, Baker JR, Harrison F, et al. The Lifestyle Engagement Activity Program (LEAP): Implementing Social and Recreational Activity into Case-Managed Home Care. *J Am Med Dir Assoc* 2015; 16: 1069–1076.

79. Low L-F, Venkatesh S, Clemson L, et al. Feasibility of LifeFul, a relationship and reablement-focused culture change program in residential aged care. *BMC Geriatr* 2018; 18: 129.

80. Lu S, Liu T, Wong GHY, et al. Health and social care service utilisation and associated expenditure among community-dwelling older adults with depressive symptoms. *Epidemiol Psychiatr Sci* 2021; 30: e10.

81. Malley JN, Towers A-M, Netten AP, et al. An assessment of the construct validity of the ASCOT measure of social care-related quality of life with older people. *Health Qual Life Outcomes* 2012; 10: 21.

82. Matos M, Bernardes SF. The Portuguese formal social support for autonomy and dependence in pain inventory (FSSADI_PAIN): A preliminary validation study. *Br J Health Psychol* 2013; 18: 593–609.

83. McGilton KS, Vellani S, Krassikova A, et al. Understanding transitional care programs for older adults who experience delayed discharge: a scoping review. *BMC Geriatr* 2021; 21: 210.

84. Meeks S, et al. Psychological and social resources relate to biomarkers of allostasis in newly admitted nursing home residents. *Aging Ment Health* 2016; 20: 88–99.

85. Milberg A, Friedrichsen M, Jakobsson M, et al. Patients' sense of security during palliative care - What are the influencing factors? *J Pain Symptom Manage* 2014; 48: 45–55.

86. Mitolo M, et al. How to enhance route learning and visuo-spatial working memory in aging: a training for residential care home residents. *Aging Ment Health* 2017; 21: 562–570.

87. Munford LA, et al. Effects of participating in community assets on quality of life and costs of care: longitudinal cohort study of older people in England. *BMJ Open* 2020; 10: e033186.

88. Murphy CM, Whelan BJ, Normand C. Formal home-care utilisation by older adults in Ireland: evidence from the Irish Longitudinal Study on Ageing (TILDA). *Health Soc Care Community* 2015; 23: 408–418.

89. Nakamura-Thomas H, Morikawa M, Moriyama Y, et al. Japanese translation and cross-cultural validation of the Adult Social Care Outcomes Toolkit (ASCOT) in Japanese social service users. *Health Qual Life Outcomes* 2019; 17: 59.

90. Naylor MD, Hirschman KB, Hanlon AL, et al. Factors Associated With Changes in Perceived Quality of Life Among Elderly Recipients of Long-Term Services and Supports. *J Am Med Dir Assoc* 2016; 17: 44–52.

91. Negron-Blanco L, de Pedro-Cuesta J, Almazan J, et al. Prevalence of and factors associated with homebound status among adults in urban and rural Spanish populations. *BMC Public Health* 2016; 16: 11.

92. Netten A, Burge P, Malley J, et al. Outcomes of social care for adults: developing a preference-weighted measure. *Health Technol Assess* 2012; 16: 1–166.

93. Netten A, Trukeschitz B, Beadle-Brown J, et al. Quality of life outcomes for residents and quality ratings of care homes: is there a relationship? *Age Ageing* 2012; 41: 512–7.

94. Neves BB, Franz RL, Munteanu C, et al. Adoption and feasibility of a communication app to enhance social connectedness amongst frail institutionalized oldest old: an embedded case study. *Info Commun Soc* 2018; 21: 1681–1699.

95. Nguyen L, Linnosmaa I, Jokimäki H, et al. Social care-related outcomes in Finland. Construct validity and structural characteristics of the Finnish ASCOT measure with older home care users. *Health Soc Care Community* 2021; 29: 712–728.

96. Nicholson E, et al. Comparing traditional-residential, personalised residential and personalised non-residential respite services: quality of life findings from an Irish population with mild-moderate intellectual disabilities. *Br J Learn Disabil* 2019; 47: 12–18.

97. Nikmat AW, Al-Mashoor SH, Hashim NA. Quality of life in people with cognitive impairment: nursing homes versus home care. *Int Psychogeriatr* 2015; 27: 815–824.

98. O'Riley AA, Van Orden KA, He H, et al. Suicide And Death Ideation in Older Adults Obtaining Aging Services. *Am J Geriatr Psychiatr* 2014; 22: 614–622.

99. Oliveira A, Nossa P, Mota-Pinto A. Assessing Functional Capacity and Factors Determining Functional Decline in the Elderly: A Cross-Sectional Study. *Acta Medica Port* 2019; 32: 654–660.

100. Ornstein K, Wajnberg A, Kaye-Kauderer H, et al. Reduction in symptoms for homebound patients receiving home-based primary and palliative care. *J Palliat Med* 2013; 16: 1048–1054.

101. Ozen B, Ceyhan O, Simsek N, et al. Frailty and quality of life in the elderly living in nursing home. *Erciyes Medical Journal* 2019; 41: 414–419.

102. Padmakar A, De Wit EE, Mary S, et al. Supported Housing as a recovery option for long-stay patients with severe mental illness in a psychiatric hospital in South India: Learning from an innovative dehospitalization process. *PLoS ONE* 2020; 15: e0230074.

103. Park JI, Han MI, Kim MS, et al. Predictors of suicidal ideation in older individuals receiving home-care services. *Int J Geriatr Psychiatry* 2014; 29: 367–376.

104. Park YH, Chang H. Effect of a health coaching self-management program for older adults with multimorbidity in nursing homes. *Patient Prefer Adherence* 2014; 8: 959–970.

105. Park EY, Park SM, Kim JH. Psychometric properties of the geriatric quality of life-dementia in older adults with dementia or mild cognitive impairment living in nursing homes. *BMC Geriatr* 2019; 19: 8.

106. Parker B, Petrou S, Underwood M, et al. Can care staff accurately assess health-related quality of life of care home residents? A secondary analysis of data from the OPERA trial. *BMJ Open* 2017; 7: e012779.

107. Pesut B, Hooper B, Jacobsen M, et al. Nurse-led navigation to provide early palliative care in rural areas: a pilot study. *BMC Palliat Care* 2017; 16: 37.

108. Phillipson L, Towers A-M, Caiels J, et al. Supporting the involvement of older adults with complex needs in evaluation of outcomes in long-term care at home programmes. *Health Expect* 2022; 25: 1453–1463.

109. Potter CM, Batchelder L, A'Court C, et al. Long-Term Conditions Questionnaire (LTCQ): initial validation survey among primary care patients and social care recipients in England. *BMJ Open* 2017; 7: e019235.

110. Pramesona BA, Taneepanichskul S. Prevalence and risk factors of depression among Indonesian elderly: A nursing home-based cross-sectional study. *Neurology Psychiatry and Brain Research* 2018; 30: 22–27.

111. Rand S, Malley J. The factors associated with care-related quality of life of adults with intellectual disabilities in England: implications for policy and practice. *Health Soc Care Community* 2017; 25: 1607–1619.

112. Rand S, Malley J, Towers AM, et al. Validity and test-retest reliability of the self-completion adult social care outcomes toolkit (ASCOT-SCT4) with adults with long-term physical, sensory and mental health conditions in England. *Health Qual Life Outcomes* 2017; 15: 163.

113. Rand SE, et al. Feasibility, factor structure and construct validity of the easy-read Adult Social Care Outcomes Toolkit (ASCOT-ER). *J Intellect Dev Disabil* 2020; 45: 119–132.

114. Rand SE, Silarova B, Towers AM, et al. Social care-related quality of life of people with dementia and their carers in England. *Health Social Care Comm*; 17. Epub ahead of print 2021. DOI: 10.1111/hsc.13681.

115. Raven MC, Kaplan LM, Rosenberg M, et al. Mobile Phone, Computer, and Internet Use Among Older Homeless Adults: Results from the HOPE HOME Cohort Study. *JMIR mHealth uHealth* 2018; 6: 15.

116. Reinhardt JP, Horowitz A, Cimarolli VR, et al. Addressing Depression in a Long-term Care Setting: A Phase II Pilot of Problem-solving Treatment. *Clin Ther* 2014; 36: 1531–1537.

117. Richardson TM, Friedman B, Podgorski C, et al. Depression and Its Correlates Among Older Adults Accessing Aging Services. *Am J Geriatr Psychiatr* 2012; 20: 346–354.

118. Olde Rikkert MGM, van der Wees PJ, Schoon Y, et al. Using patient reported outcomes measures to promote integrated care. *Int J Integr Care* 2018; 18: 1–7.

119. Saunders S, Weiss ME, Meaney C, et al. Examining the course of transitions from hospital to home-based palliative care: A mixed methods study. *Palliat Med* 2021; 35: 1590–1601.

120. Sheffield C, Smith CA, Becker M. Evaluation of an Agency-Based Occupational Therapy Intervention to Facilitate Aging in Place. *Gerontologist* 2013; 53: 907–918.

121. Shippee TP, et al. Resident- and facility-level predictors of quality of life in long-term care. *Gerontologist* 2015; 55: 643–655.

122. Siegert R, Selman L, Higginson IJ, et al. A Psychometric Evaluation of the Functional Assessment of Chronic Illness Therapy-Palliative Care (FACIT- Pal) Scale With Palliative Care Samples in Three African Countries. *J Pain Symptom Manage* 2014; 48: 983–991.

123. Siette J, Georgiou A, Jorgensen M, et al. Integrating social engagement instruments into Australian community aged care assessments to enhance service provision. *Health Soc Care Community* 2018; 26: 810–818.

124. Siette J, Georgiou A, Brayne C, et al. Social networks and cognitive function in older adults receiving home- and community-based aged care. *Arch Gerontol Geriatr* 2020; 89: 104083.

125. Simning A, Caprio TV, Seplaki CL, et al. Rehabilitation Providers' Prediction of the Likely Success of the SNF-to-Home Transition Differs by Discipline. *J Am Med Dir Assoc* 2019; 20: 492–496.

126. Sirey JA, Greenfield A, DePasquale A, et al. Improving engagement in mental health treatment for home meal recipients with depression. *Clin Interv Aging* 2013; 8: 1305–1312.

127. Siverova J, Buzgova R. The effect of reminiscence therapy on quality of life, attitudes to ageing, and depressive symptoms in institutionalized elderly adults with cognitive impairment: A quasi-experimental study. *Int J Ment Health Nurs* 2018; 27: 1430–1439.

128. Smelson DA, et al. Integrating permanent supportive housing and co-occurring disorders treatment for individuals who are homeless. *J Dual Diagn* 2016; 12: 193–201.

129. Smith N, Towers AM, Palmer S, et al. Being occupied: supporting "meaningful activity' in care homes for older people in England. *Ageing Soc* 2018; 38: 2218–2240.

130. Smith GM, Calton BA, Rabow MW, et al. Comparing the Palliative Care Needs of Patients Seen by Specialty Palliative Care Teams at Home Versus in Clinic. *J Pain Symptom Manage* 2021; 62: 28–38.

131. Spoorenberg SLW, Wynia K, Uittenbroek RJ, et al. Effects of a population-based, person-centred and integrated care service on health, wellbeing and self-management of community-living older adults: A randomised controlled trial on Embrace. *PloS One* 2018; 13: e0190751.

132. Spoorenberg SL, Reijneveld SA, Uittenbroek RJ, et al. Health-Related Problems and Changes After 1 Year as Assessed With the Geriatric ICF Core Set (GeriatrICS) in Community-Living Older Adults Who Are Frail Receiving Person-Centered and Integrated Care From Embrace. *Arch Phys Med Rehabil* 2019; 100: 2334–2345.

133. Stergiopoulos V, Gozdzik A, Misir V, et al. Effectiveness of Housing First with Intensive Case Management in an Ethnically Diverse Sample of Homeless Adults with Mental Illness: A Randomized Controlled Trial. *PLoS One* 2015; 10: 21.

134. Szanton SL, Xue QL, Leff B, et al. Effect of a Biobehavioral Environmental Approach on Disability Among Low-Income Older Adults A Randomized Clinical Trial. *JAMA Intern Med* 2019; 179: 204–211.

135. Tay RY, Choo RWK, Ong WY, et al. Predictors of the final place of care of patients with advanced cancer receiving integrated home-based palliative care: a retrospective cohort study. *BMC Palliat Care* 2021; 20: 164.

136. Taylor HO, et al. Assessing social isolation: pilot testing different methods. *J Gerontol Soc Work* 2016; 59: 228–233.

137. Tazopoulou E, Miljkovitch R, Truelle JL, et al. Rehabilitation following cerebral anoxia: An assessment of 27 patients. *Brain Inj* 2016; 30: 95–103.

138. Toles M, Preisser JS, Colón-Emeric C, et al. Connect-Home transitional care from skilled nursing facilities to home: A stepped wedge, cluster randomized trial. *J Am Geriatr Soc* 2023; 71: 1068–1080.

139. Tosangwarn S, Clissett P, Blake H. Predictors of depressive symptoms in older adults living in care homes in Thailand. *Arch Psychiatr Nurs* 2018; 32: 51–56.

140. Towers A-M, Holder J, Smith N, et al. Adapting the adult social care outcomes toolkit (ASCOT) for use in care home quality monitoring: conceptual development and testing. *BMC Health Serv Res* 2015; 15: 304.

141. Towers A-M, Smith N, Palmer S, et al. The acceptability and feasibility of using the Adult Social Care Outcomes Toolkit (ASCOT) to inform practice in care homes. *BMC Health Serv Res* 2016; 16: 523.

142. Travers C, Barlett HP. Silver Memories: implementation and evaluation of a unique radio program for older people. *Aging Menl Health* 2011; 15: 169–177.

143. Turnpenny A, et al. Developing an easy read version of the Adult Social Care Outcomes Toolkit (ASCOT). *J Appl Res Intellect Disabil* 2018; 31: e36–e48.

144. Uittenbroek RJ, van Asselt ADI, Spoorenberg SLW, et al. Integrated and Person-Centered Care for Community-Living Older Adults: A Cost-Effectiveness Study. *Health Serv Res* 2018; 53: 3471–3494.

145. Valdovinos C, Ingle MP, Ford KL, et al. Patient Portals to Support Palliative and End-of-Life Care: Scoping Review. *J Med Internet Res* 2021; 23: e28797.

146. van Leeuwen KM, Malley J, Bosmans JE, et al. What can local authorities do to improve the social care-related quality of life of older adults living at home? Evidence from the Adult Social Care Survey. *Health & place* 2014; 29: 104–13.

147. van Leeuwen KM, Bosmans JE, Jansen APD, et al. Comparing Measurement Properties of the EQ-5D-3L, ICECAP-O, and ASCOT in Frail Older Adults. *Value Health* 2015; 18: 35–43.

148. Waldrop D, Meeker MA, Kutner JS. The developmental transition from living with to dying from cancer: Hospice decision making. *J Psychosoc Oncol* 2015; 33: 576–598.

149. Walshe C, Dodd S, Hill M, et al. How effective are volunteers at supporting people in their last year of life? A pragmatic randomised wait-list trial in palliative care (ELSA). *BMC Med* 2016; 14: 12.

150. Watkins L, Hall C, Kring D. Hospital to home: a transition program for frail older adults. *Prof Case Manag* 2012; 17: 117–5.

151. Whitehead PJ, Golding-Day MR, Belshaw S, et al. Bathing adaptations in the homes of older adults (BATH-OUT): results of a feasibility randomised controlled trial (RCT). *BMC Public Health* 2018; 18: 1293.

152. Wilberforce M, Challis D, Davies L, et al. The preliminary measurement properties of the person-centred community care inventory (PERCCI). *Qual Life Res* 2018; 27: 2745–2756.

153. Wilner P, et al. Group-based cognitive-behavioural anger management for people with mild to moderate intellectual disabilities: cluster randomised controlled trial. *Br J Psychiatry* 2013; 203: 288–296.

154. Wong AKC, Wong FKY, Chow KKS, et al. Effect of a Mobile Health Application With Nurse Support on Quality of Life Among Community-Dwelling Older Adults in Hong Kong: A Randomized Clinical Trial. *JAMA Netw Open* 2022; 5: e2241137.

155. Woolham J, Daly G, Sparks T, et al. Do direct payments improve outcomes for older people who receive social care? Differences in outcome between people aged 75+ who have a managed personal budget or a direct payment. *Ageing Soc* 2017; 37: 961–984.

156. Xiang W, Cheng Y, Li Z, et al. Cross-cultural adaptation and validation of the Groningen Frailty Indicator in Chinese nursing home residents. *Aging Clin Exp Res* 2020; 32: 1035–1042.

157. Yang W, Forder J, Nizalova O. Measuring the productivity of residential long-term care in England: methods for quality adjustment and regional comparison. *Eur J Health Econ: HEPAC : health economics in prevention and care* 2017; 18: 635–647.

158. Yao CT, Yang YP, Chen YC. Positive effects of art therapy on depression and self-esteem of older adults in nursing homes. *Soc Work Health Care* 2019; 58: 324–338.

159. Ysseldyk R, Haslam SA, Haslam C. Abide with me: religious group identification among older adults promotes health and well-being by maintaining multiple group memberships. *Aging Ment Health* 2013; 17: 869–879.
