# Supplementary figures and images for: Patient-reported outcomes in integrated health and social care: A scoping review
Source: JRSM Open. 2024 Mar 24;15(3):20542704241232866. doi: 10.1177/20542704241232866 (PMC10962043; doi:10.1177/20542704241232866)

**Supplementary Appendix 3. Year of publication of the included articles (2010 – 2023)**

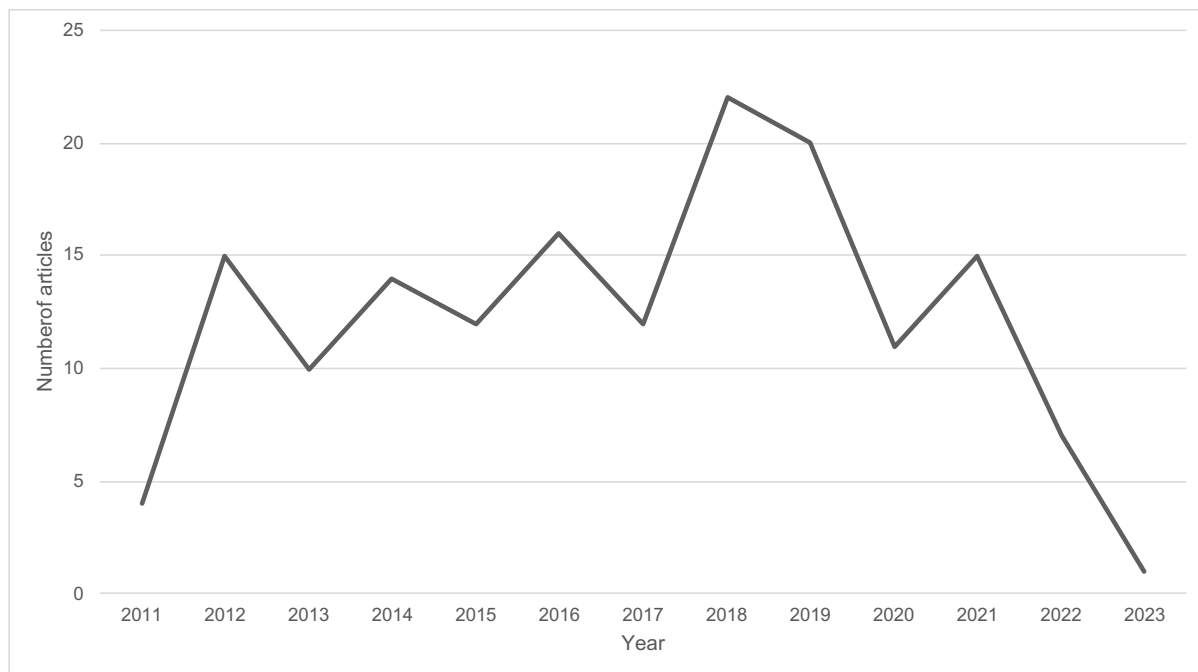

Supplement: sj-pdf-3-shr-10.1177_20542704241232866 - Supplemental material for Patient-reported outcomes in integrated health and social care: A scoping review [file sj-pdf-3-shr-10.1177_20542704241232866.pdf]
