# Supplementary material for: Patient-reported outcomes in integrated health and social care: A scoping review
Source: JRSM Open. 2024 Mar 24;15(3):20542704241232866. doi: 10.1177/20542704241232866 (PMC10962043; doi:10.1177/20542704241232866)
Supplement: sj-pdf-4-shr-10.1177_20542704241232866 - Supplemental material for Patient-reported outcomes in integrated health and social care: A scoping review [file sj-pdf-4-shr-10.1177_20542704241232866.pdf]

Supplementary Appendix 4. Number of articles utilising the PRO measures identified in this scoping review (n = 216)

PROM	N articles	PROM	N articles	PROM	N articles
EQ5D (All Versions)	28	Overall QOL (Bowling 1995)	1	Philadelphia Geriatric Center Morale Scale (PGCMS)	1
Geriatric Depression Scale (GDS)	25	ICT-Brief	1	Cognitive Failures Questionnaire (CFQ)	1
ASCOT (All versions)	5	Aged Care Fit	1	Brief-Cope (B-Cope)	1
PHQ-9	12	NHP-5 (Pain)	1	Social Identification and Satisfaction (SIS)	1
SF-36	8	Personal Outcomes Scale	1	Short Physical Performance Battery (SPPB)	1
Centre for Epidemiologic Studies Depression Scale (CES-D)	7	Assessment of QoL Scale	1	Cornell Scale for Depression	1
SF-12	7	OAPAM	1	Disability Assessment for Dementia (DAD)	1
Barthel Index	7	OAMA	1	Cohen-Mansfield Agitation Inventory (CMAI)	1
Edmonton Symptom Assessment System (ESAS)	6	Face Anxiety Scale	1	Control and autonomy subscale of the (CASP-12)	1
ADL Limitations (Katz 1963)	6	Smile-o-meter	1	Social Support Scale of Medical Outcomes Study (SSS-MOS)	1
QoL in Alzheimer's Disease (QOL-AD)	6	PEG-3 Item Pain Scale	1	FSSADI_PAIN	1
UCLA Lonelieness Scale	6	MANSA	1	Adult Hope Scale	1
WHOQOL	6	Brief Symptom Index	1	Rating Anxiety in Dementia (RAID)	1
Multi-dimensional Scale of Perceived Social Support (MSPSS)	6	Penn State Worry Questionnaire (PSWQ)	1	Sense of security in Care instrument for patients	1
ICECAP-O	5	Lawton IADL	1	General Self-Efficacy Scale	2
PROMIS (all scales)	5	Health Care Empowerment Questionnaire (HCEQ)	1	Experiences in Close Relationships	1
Rosenberg Self-Esteem Scale (RSES)	4	Multi-dimensional Outcome Expectations for Exercise Scale (MOEES)	1	Spatial Anxiety Scale (SA)	1
Hospital Anxiety and Depression Scale (HADS)	4	Balance Confidence Scale	1	Spatial Self-Efficacy Questionnaire (SSEQ)	1
Lubben Social Network Scale (LSNS)	4	Health-o-meter	1	Sense of Direction and Spatial Representation Questionnaire (SDSRQ)	1
Patient Activation Measure	3	Older Peoples Quality of life Brief Questionnaire (OPQoL-Brief)	1	Attitude to Environmental Tasks Questionnaire (AETQ)	1
African Palliative Outcome Scale (POS)	3	Kessler Psychological Distress Scale	1	Single item QoL rating	1
EORTC QLQ (All versions)	3	Exeter Identity Transition Scales (EXITS)	1	Symptom Bother Scale	1
Duke Social Support Index (Koenig et al 1993)	3	Sheldon-Cohen Perceived Stress Scale	1	Friendship Scale translated into Malay	1

Brief Pain Inventory (BPI)	3	Person-centred community care inventory (PERCCI)	1	EUROHIS-QOL (WHO-8)	1
Groningen Frailty Indicator (GFI)	3	Provocation Index	1	Paykel Suicide Scale	1
Connor-Davidson Resilience Scale (CDRS)	2	Profile of Anger Coping Skills	1	Goldberg Anxiety Scale (GAS)	1
Social Support Questionnaire	2	Glasgow Depression and Anxiety Scales (GDS/GAS)	1	Alcohol Use Disorders Identification Test (AUDIT-C)	1
Canadian Occupational Performance Measure	2	Comprehensive Quality of Life Scale - Intellectual Disability (ComQoL-ID)	1	Scale for Suicidal Ideation	1
ACCOM	2	De Jong Gierveld Scale	1	Health Assessment Questionnaire (HAQ)	1
Perceived Stress Scale	2	Quality of Life GRS	1	Chronic Disease Self-Management Program Questionnaire (CDSMPQ)	1
Pearlin & Schooner Mastery	2	Health Global Rating Scale	1	Bayliss Burden of Morbidity scale	1
Epworth Sleepiness Scale	2	Personal Wellbeing Index - Intellectual Disability (PWI-ID)	1	Self-efficacy for Managing Chronic Disease scale	1
Lehman's Brief QoL Interview	2	WEll-being Star	1	Long Term Conditions Questionnaire (LTCQ)	1
European Addiction Severity Index	2	Measure Yourself Medical Outcomes Profile (MYMOP)	1	Hamilton Rating Scale for Depression (HAM-D)	1
Short Falls Efficacy Scale	2	Measure Yourself Concerns and Wellbeing (MYCAW)	1	QoL questionnaire	1
mMOSS-SS	2	Cut-Annoyed-Guilt-Eye-Opener (CAGE) questionnaire	1	FACT-G	1
Warwick-Edinburgh Mental Health and Wellbeing Measure	2	Wong Baker Pain Rating Scale (WBPRS)	1	FACIT-Pal	1
Rockwood Clinical Frailty Scale	2	Urban life stress (Urban Life Stress Scale, ULSS)	1	Eastern Cooperative Oncology Group scale	1
Geriatric Anxiety Inventory and GAI-Short Form	2	distress tolerance (Distress Tolerance Scale)	1	Montgomery Asberg Depression Rating Scale (MADRS)	1
WHODAS	2	perceived social support (Interpersonal Support Evaluation List 12)	1	Suicide Risk Assessment	1
McGill QoL Questionnaire	2	PTSD symptoms (Primary Care Post-Traumatic Stress Disorder screener, PC-PTSD)	1	The Attitude to Aging Questionnaire (AAQ)	1
Satisfaction with Life Scale	2	Brief STAI	1	Behaviour and Symptom Identification Scale (BASIS-32)	1
Visual Analogue Scale (VAS)	2	Life Satisfaction Scale	1	Addition Severity Index (ASI)	1
General Health Questionnaire (GHQ-12)	2	Duke Life Event Scale (Blazer at el 1987)	1	Groningen Well-being Indicator (GWI)	1
Life Orientation Test - Revised	2	Family Adaptation, Partnership, Growth, Affection, andResolve (APGAR)	1	Self-Management Ability Scale (SMAS-30)	1
Louisville Older Persons Stress Scale	2	Apathy Evaluation Scale	1	Partners in Health Scale for Older Adults (PIH-OA)	1

Quality of Life after Traumatic Brain Injury (QoLIBRI)	2	Instrumental Activities of Daily Life (IADL)	1	INTERMED for the Elderly Self Assessment (IM-E-SA)	1
GAD-7	1	Australian Community Participation Questionnaire (ACPQ)	1	Community Integration Scale (CIS)	1
Self-reported QoL Scale(McConkey, Bunting, Ferry, Garcia-Iriarte & Stevens, 2013)	1	Geriatric Quality of Life Scale-Dementia (GQOL-D)	1	Quality of Life Index (QoLI-20)	1
Visual Analogue Scale (VAS)	1	The Alcohol, Smoking, and Substance Involvement Screening Test (ASSIST)	1	Substance disorder screener	1
WHO-5	1	Community Integration Questionnaire	1	GAIN short screener	1
Integrated Palliative Outcome Scale for Dementia (IPOS-Dem)	1	Disability Rating Scale	1	Colorado Symptom Index	1
BURS	1	Palliative Care Screening Tool	1	Patient Compentency Rating Scale (PCRS)	1
RS	1	Mental Adjustment to Cancer Scale (MAC)	1	Bermont Vost Alexithymia Questionnaire	1
Post-discharge Coping Difficulty Scale (PDCDS)	1	Desire for Death (DDRS)	1	Internalised Stigma of Living in a Care Home Scale	1
Utrecht Symptom Diary-4 Dimensional	1	Family of Origin Instability Scale	1	Coping Strategies Inventory Short Form	1
Empowerment Scale (Robers 1997)	1	Helzer Conduct Disorder Scale	1	Single item on Loneliness	1
Process of Personal Recovery (Neil 2009)	1	Outcomes Star Motivational Chart Tool (Age UK)	1	Ego-integrity despair measure (Dezutter et al 2016 adapted from Van Hiel & Vansteenkiste 2009)	1
BSRS-5	1	Quality of Life in Life-Threatening Illness-Family Carer Version (QOLLTI-F)	1	Pittsburgh Sleep Quality Index (PSQI)	1
Life Satisfaction Index	1	Palliative Outcome Scale (POS)	1	Memorial Symptom Assessment Scale	1
McArthur Scale of subjective social status	1	Assessment of symptom burden using the Minimal Documentation System for Palliative Medicine (MIDOS)	1	Palliative Performance Scale	1
PELI-NH	1	Chinese Citizen Health Literacy Questionnaire	1	EQ6D	1
Nottingham Extended Activities of Daily Living	1	Exercise of Self-Care Agency Scale	1	Palliative Care outcome scale	1
PFSSADI	1	SSRS (measure degree of social support)	1	Integrated Palliative Care Outcome Scale	1
Community Dependency Index	1	Happiness Subscale of the Center for Epidemiological Studies	1	interRAI self-report survey on nursing home quality of life	1
Morriston OT Outcome Measure	1	Brief Symptom Inventory or BSI Anxiety Scale	1	Brief Relationship Satisfaction Scale	1
ENRICHD Social Support Instrument	1	Neff Scale for Self-Compassion	1	Missoula-VITAS Quality of Life Index	1
BADLS	1	Social Support Inventory	1	Pain and Symptom Assessment Record	1
QOL Scales for Nursing Home Residents	1	BMMRS	1	Rated Health Question (SRH; Idler & Benyamini, 1997)"	1
Self-perceived health (Robine)	1	Asian Values Scale Revised	1	Care Transitions Measure – 15 (CTM-15)	1

Collett-Lester Fear of Death Scale (CL-FODS)	1	Hospice Attitudes Scale - Modified	1	Life Space Assessment	1
Modified Emanuel Medical Directives (MEMD)	1	UCLA Social Support Inventory	1		
