# Supplementary material for: Patient-reported outcomes in integrated health and social care: A scoping review
Source: JRSM Open. 2024 Mar 24;15(3):20542704241232866. doi: 10.1177/20542704241232866 (PMC10962043; doi:10.1177/20542704241232866)
Supplement: sj-pdf-5-shr-10.1177_20542704241232866 - Supplemental material for Patient-reported outcomes in integrated health and social care: A scoping review [file sj-pdf-5-shr-10.1177_20542704241232866.pdf]

Supplementary Appendix 5. PRO Measures classified by construct of interest (n = 216)

PRO Measure	Construct	Functional Status	Psychological Status	Symptom Burden	Quality of Life	Other
ACCOM	Uses a standardized set of instruments to measures functional, health, and social care-related quality of life (SCRQoL) among older people receiving services at home.				1	
Addition Severity Index (ASI)	To produce a problem severity profile of each patient through an analysis of six general areas		1			
ADL Limitations (Katz 1963)	To assess functional status as a measurement of the client's ability to perform activities of daily living independently	1				
Adult Hope Scale	The relationship among low, moderate, and high levels of hopelessness, all-cause and cause-specific mortality, and incidence of myocardial infarction (MI) and cancer in a population-based sample of middle-aged men		1			
African Palliative Outcome Scale (POS)	To measure palitative needs of patients and their families				1	
Aged Care Fit	To measure the interaction between an individual and their Measure of person-environment fit (the interaction between an individual and their long term care environment) - looks at positive affect and adaptive behaviour	1				
Alcohol Use Disorders Identification Test (AUDIT-C)	To assess hazardous and harmful alcohol consumption and screen for problem drinking which is at an earlier or milder stage.		1			
Apathy Evaluation Scale	To measure the severity of apathy (including the dimensions of emotional apathy, cognitive apathy, behavioural apathy)		1			
ASCOT (All versions)	Designed to measure the aspects of an individual's quality of life that can be affected by social care.				1	
Asian Values Scale - Revised	To measure Asian values adherence at the cultural level					1
Assessment of QoL Scale	Health-Related Quality of Life - HRQL (incl. health status)				1	
Assessment of symptom burden using the Minimal Documentation System for Palliative Medicine (MIDOS)	Physical and cognitive impairment of the patients may impede the use of standardized questionnaires and documentation systems in palliative care setting. We developed a minimal documentation system (MIDOS) for the specific requirements in this setting			1		

PRO Measure	Construct	Functional Status	Psychological Status	Symptom Burden	Quality of Life	Other
Attitude to Environmental Tasks Questionnaire (AETQ)	It contains seven items about the use of spatial representation strategies and spatial abilities.		1			
Australian Community Participation Questionnaire (ACPQ)	To measure community participation		1			
BADLS	To assess residual capacities and change of abilities in people with dementia	1				
Balance Confidence Scale (activities-specific?)	A self-report measure of balance confidence in performing various activies without losing balance or experiencing a sense of unsteadyness	1				
Barthel Index	Measure performance in activities of daily living (ADL)	1				
Bayliss Burden of Morbidity scale	Measuring generic functioning and well-being and disease-specific health outcomes	1				
Behaviour and Symptom Identification Scale (BASIS-32)	Signs and symptoms			1		
Bermont Vorst Alexithymia Questionnaire	Measures two affective features in addition to three cognitive features of alexithymia, based on which two high-order structures of cognitive and affective components can be formed.		1			
Brief Multi-Dimensional Measure of Religiousness/Spirituality (BMMRS)	A paper-pencil, self-report measure of different dimensions or facets of religiousness and spirituality		1			
Brief Pain Inventory (BPI)	To assess the severity and impact of pain experienced by individuals			1		
Brief Relationship Satisfaction Scale	A measure of general relationship satisfaction		1			
Brief STAI	Measure of trait and state anxiety		1			
Brief Symptom Index	To assess psychological distress and psychiatric disorders		1			
Brief Symptom Inventory or BSI Anxiety Scale	Measures a broad range of psychological symptoms and distress in individuals		1			
Brief-Cope (B-Cope)	Measures a broad range of psychological symptoms		1			
BSRS-5	To assess psychological wellbeing and to detect the presence of psychological symptoms		1			
BURS	Psychological resilience among palliative patients with advanced cancer		1			

PRO Measure	Construct	Functional Status	Psychological Status	Symptom Burden	Quality of Life	Other
Canadian Occupational Performance Measure	To assess occupational performance problems (self-care, productivity & leisure) using the client's own words	1				
Care Transitions Measure – 15 (CTM-15)	Measures patient preparedness at discharge					1
Centre for Epidemiologic Studies Depression Scale (CES-D)	Measure of depression		1			
Chinese Citizen Health Literacy Questionnaire	Collects information on how chinese citizens find, understand and use health information, and how they manage their health and interact with the health system and healthcare providers.					1
Chronic Disease Self-Management Program Questionnaire (CDSMPQ)	A standardized self-management intervention for patients with various chronic diseases.				1	
Cognitive Failures Questionnaire (CFQ)	Psychological functioning (incl. coping)		1			
Cohen-Mansfield Agitation Inventory (CMAI)	To assess the frequency of manifestations of agitated behaviors in elderly persons with cognitive impairment		1			
Collett-Lester Fear of Death Scale (CL-FODS)	To assess an individual's level of fear or anxiety specifically related to the fear of death.		1			
Colorado Symptom Index	To assess frequency of positive mood and cognitive symptoms.		1			
Community Dependency Index	A Standardised Assessment of Need and Occupational Therapy	1				
Community Integration Questionnaire	Measure levels of community integration	1				
Community Integration Scale (CIS)	Social functioning (incl. work) and physical functioning	1				
Community Resources Support (CRS) Questionnaire	Assesses perceive available resources regarding end-of-life healthcare planning at the community level.					1
Comprehensive Quality of Life Scale - Intellectual Disability (ComQoL-ID)	Quality of life in ID		1			
Connor-Davidson Resilience Scale (CDRS)	To measure stress coping ability		1			
Control and autonomy subscale of the (CASP-12)	A measure of quality of life (QoL) in older ages - The measures of control, autonomy, self-realization and pleasure in the CASP-12.				1	
Coping Strategies Inventory Short Form	To categorize coping responses based on coping target and directionality of response		1			

PRO Measure	Construct	Functional Status	Psychological Status	Symptom Burden	Quality of Life	Other
Cornell Scale for Depression	To assess signs and symptoms of major depression in demented patients.		1			
Cut-annoyed-guilt-eye-opener (CAGE) questionnaire	Used to test for alcohol abuse and dependence in adults.		1			
De Jong Gierveld Scale	A measurement instrument for overall, emotional, and social loneliness.		1			
Desire for Death (DDRS)	To determine the prevalence, severity and remission of desire for hastened death.		1			
Disability Assessment for Dementia (DAD)	To quantitatively measure functional abilities in activities of daily living (ADL) in individuals with cognitive impairments such as dementia	1				
Disability Rating Scale	To chart the progress of severe head injury (HI) patients	1				
distress tolerance (Distress Tolerance Scale)	Measuring the perceived capacity to tolerate distress from a multidimensional framework		1			
Duke Life Event Scale (Blazer at el 1987)	Assesses caregivers' perceived levels of both support and stress associated with people in the identified family and non-family relationships		1			
Duke Social Support Index (DSSI, Koenig et al 1993)	Used to measure social support of the elderly		1			
Eastern Cooperative Oncology Group scale	To assess the functional status of cancer patients	1				
Edmonton Symptom Assessment System (ESAS)	To assist in the assessment of nine common symptoms in palliative care patients (revised 2011)			1		
ego-integrity despair measure (Dezutter et al 2016 adapted from Van Hiel & Vansteenkiste 2009)	To measure ego integrity and despair as two separate dimensions		1			
Empowerment Scale (Robers 1997)	To assess empowerment		1			
ENRICHD Social Support Instrument	To measure the participant’s range of social support in their life		1			
EORTC QLQ (All versions)	To assess quality of life in a wide range of cancer patient populations				1	
Epworth Sleepiness Scale	To measure a subject's usual level of daytime sleepiness or average sleep propensity	1				
EQ5D (All Versions)	Health-Related Quality of Life - HRQL (incl. health status) and Utility				1	

PRO Measure	Construct	Functional Status	Psychological Status	Symptom Burden	Quality of Life	Other
EQ6D	Health-Related Quality of Life - HRQL (incl. health status) Utility				1	
WHO-8	A quality of life assessment - composed of eight items (overall QOL, general health, energy, daily life activities, esteem, relationships, finances, and home)				1	
European Addiction Severity Index	a) to assess the problem severity of the interviewee, and b) for periodic repeated administrations to monitor and quantify change in problems commonly associated to substance abuse.		1			
Exercise of Self-Care Agency Scale	An instrument to measure exercise of self-care agency		1			
Exeter Identity Transition Scales (EXITS)	Examine group membership		1			
Experiences in Close Relationships	36-item questionnaires on adult romantic attachment style, comprising two scales assessing attachment anxiety and avoidance.		1			
Face Anxiety Scale	Self-report measure of state anxiety in intensive care patients that is easy to administer and imposes minimal respondent burden		1			
FACIT-Pal	To assess quality of life for persons with advanced or life-limiting illness				1	
FACT-G	To measure the quality of life in people with cancer				1	
Family Adaptation, Partnership, Growth, Affection, and Resolve (APGAR)	Assess adult satisfaction with social support from the family		1			
Family of Origin Instability Scale	A scale in which respondents provide a retrospective assessment of the family in which they were raised		1			
Friendship Scale translated into Malay	Assesses the quality of children's and early adolescents' relationships with their best friends according to five conceptually meaningful aspects of the friendship relation.		1			
FSSADI_PAIN	Assesses older adults' perceptions of the frequency with which they have received pain-related social support from staff at their institution.		1			
GAD-7	To identify probable cases of Generalized Anxiety Disorder (GAD) and assess symptom severity in GAD		1			
GAIN short screener	3–5 minute screener to quickly identify those who would have a disorder based on the full 60–120 minute GAIN and triage the problem and kind of intervention they are likely to need along four dimensions					1

PRO Measure	Construct	Functional Status	Psychological Status	Symptom Burden	Quality of Life	Other
General Health Questionnaire (GHQ-12)	Screening instrument to detect psychiatric disorders in community settings and non-psychiatric clinical settings, such as primary care or general practice		1			
General Self-Efficacy Scale	To assess a general sense of perceived self-efficacy with the aim in mind to predict coping with daily hassles as well as adaptation after experiencing all kinds of stressful life events		1			
Geriatric Anxiety Inventory and GAI-Short Form	To measure anxiety in older people		1			
Geriatric Depression Scale (GDS)	Screening test for depression in elderly people		1			
Geriatric Quality of Life Scale-Dementia (GQOL-D)	To compare the QOL of dementia patients with that of a control group.				1	
Glasgow Depression and Anxiety Scales (GDS/GAS)	Used for evaluating behaviours related to depression in people with a learning disability and ID		1			
Goldberg Anxiety Scale (GAS)	Developed specifically to find out the probability that a state of anxiety or depression will occur.		1			
Groningen Frailty Indicator (GFI)	To assess the physical, cognitive, social, and psychological domains	1				
Groningen Well-being Indicator (GWI)	Measures well-being		1			
Hamilton Rating Scale for Depression (HAM-D)	To measure severity and treatment effects in depressed patients		1			
Happiness Subscale of the Center for Epidemiological Studies	Measures a Continuum from Well-Being to Depression.		1			
Health Assessment Questionnaire (HAQ)	Physical functioning, Activities of Daily Living and Signs and symptoms	1				
Health Care Empowerment Questionnaire (HCEQ)	Measures the degree of individual empowerment in relation to personal health care and services.		1			
Health-o-meter	To assess the interaction between comorbidities and self-reported health status as the basis for preventive intervention in care of the elderly	1				
Helzer Conduct Disorder Scale	To allow for measurement of conduct disorder (CD)		1			
Hospice Attitudes Scale - Modified	Assesses an individual's attitudes toward hospice care. Specifically, it aims to measure attitudes and beliefs relate					1
Hospital Anxiety and Depression Scale (HADS)	To detect states of anxiety and depression		1			

PRO Measure	Construct	Functional Status	Psychological Status	Symptom Burden	Quality of Life	Other
ICECAP-O	Measure of wellbeing for use in ecomic evaluation (capability)		1			
ICT-Brief	Self-assessment/caregiver-assisted tool to screen the health needs of older adults	1				
Instrumental Activities of Daily Life (IADL)	To assess everyday functional competence	1				
Integrated Palliative Care Outcome Scale	To measure palliative needs of patients and their families (domains incl. physical psych symptoms, social/spiritual symptoms, communication, informational needs and practical concerns.			1		
Integrated Palliative Outcome Scale for Dementia (IPOS-Dem)	Caregiver-reported measure to detect symptoms and problems in dementia.			1		
INTERMED for the Elderly Self Assessment (IM-E-SA)	Assesses case complexity and health care needs as perceived by older adults themselves.					1
Internalised Stigma of Living in a Care Home Scale	To assess internalized stigma in older adults living in care settings		1			
interRAI self-report survey on nursing home quality of life	To determine how persons served in a variety of programs and settings experience day-to-day life and assess the services they receive.					1
Kessler Psychological Distress Scale	To assess nonspecific psychological distress in epidemiologic surveys		1			
Lawton IADL	To assess everyday functional competence	1				
Lehman's Brief QoL Interview	To assess the life circumstances of persons with severe mental illnesses both in terms of what they actually do and experience ("objective" quality of life) and their feelings about these experiences ("subjective" quality of life)				1	
Life Orientation Test - Revised	To measure individual differences in optimism versus pessimism		1			
Life Satisfaction Index	Assesses an individual's cognitive judgment of their satisfaction with their life as a whole		1			
Life Satisfaction Scale	To measure personal life satisfaction		1			
Life Space Assessment	The Life-Space Assessment a5-item scale in 5 levels of life-space, with higher scores indicating greater function.	1				
Long Term Conditions Questionnaire (LTCQ)	To understand the impact of long-term health conditions on people’s lives, and to find out what support those people want or need			1		

PRO Measure	Construct	Functional Status	Psychological Status	Symptom Burden	Quality of Life	Other
Louisville Older Persons Stress Scale	To measure stress - designed for older adults on the basis of extensive pretesting with older populations.		1			
Lubben Social Network Scale (LSNS)	A self-report measure of social engagement including family and friends (correlates with mortality, all case hospitalization, health behaviors, depressive symptoms, and overall physical health).		1			
MANSA	A brief instrument used for assessing quality of life focusing on satisfaction with life as a whole and with life domains.				1	
McArthur Scale of subjective social status	A single-item measure that assesses a person's perceived rank relative to others in their group.		1			
McGill QoL Questionnaire	Evaluates the physical condition's impact on the quality of life, rather than on the intensity of symptoms.				1	
Measure Yourself Concerns and Wellbeing (MYCAW)	To evaluate complementary therapies in cancer support care.		1			
Measure Yourself Medical Outcomes Profile (MYMOP)	Signs and symptoms, pyschological functioning (incl. coping) and activities of daily living - To assess the sensitivity to within person change over time		1			
Memorial Symptom Assessment Scale	- To provide multidimensional information about a diverse group of common symptoms in the cancer population			1		
Mental Adjustment to Cancer Scale (MAC)	Used to assess psychological response to cancer.		1			
Missoula-VITAS Quality of Life Index	A tool to assess subjective quality of life among people living with advanced, life-threatening illness.				1	
mMOSS-SS	To measure social support and supports, particularly in multidimensional geriatric assessments and specifically in older women with breast cancer.		1			
Modified Emanuel Medical Directives (MEMD)	To assess life-sustaining treatment preference.					1
Montgomery Asberg Depression Rating Scale (MADRS)	To detect change during antidepressant medicine trials.		1			
Morriston OT Outcome Measure	It measures individual change in occupational performance.	1				
MSPSP	A 12-item measure that assesses three domains of social support–family, friends and significant other.		1			

PRO Measure	Construct	Functional Status	Psychological Status	Symptom Burden	Quality of Life	Other
Multi-dimensional Outcome Expectations for Exercise Scale (MOEES)	A scale assessing outcome expectations, physical activity, self-efficacy, and health status.	1				
Multi-dimensional Scale of Perceived Social Support (MSPSS)	To assess perceived adequacy of social support from three sources: family, friends, and significant other		1			
Neff Scale for Self-Compassion	Measures the three main components of self-compassion on separate subscales (self-kindness versus self-judgment, common humanity versus isolation, and mindfulness versus over-identification).		1			
NHP-5 (Pain)	Assesses five dimensions of health status: mobility, self-care, usual activities, pain/discomfort, and anxiety/depression.			1		
Nottingham Extended Activities of Daily Living	To assess stroke patients living in the community	1				
OAMA	To aid in the assessment of pyschological abuse of older adults		1			
OAPAM	An assessment tool designed to assist medical providers and social service practitioners in measuring whether their clients experience any form of mistreatment or abuse - is one scale of the Older Adult Mistreatment Assessment (OAMA)		1			
Older Peoples Quality of life Brief Questionnaire (OPQoL-Brief)	To measure Quality Of Life in Older People				1	
Outcomes Star Motivational Chart Tool (Age UK)	A unique outcomes measurement tool to measure and summarise change across a range of services.		1			
Overall QOL (Bowling 1995)	A measurement of quality of life (QoL) in social care.				1	
Pain and Symptom Assessment Record	To assess patients' pain and symptoms in order to facilitate communication among health care professionals within various health care settings.			1		
Palliative Care outcome scale	To help clinical practitioners meet people's palliative care needs. 2) For use in research studies to monitor outcomes				1	
Palliative Care Screening Tool	Aims to address the multidimensional needs of patients and families.	1				
Palliative Outcome Scale (POS)	To measure patients' physical symptoms, psychological, emotional and spiritual, and information and support needs.				1	

PRO Measure	Construct	Functional Status	Psychological Status	Symptom Burden	Quality of Life	Other
Palliative Performance Scale	A functional assessment of a patient's ambulation, activity level, evidence of disease, self-care, food/fluids intake, and level of consciousness.	1				
Partners in Health Scale for Older Adults (PIH-OA)	Assessing the self-management knowledge and behaviour of older adults.		1			
Patient Activation Measure	Assesses the knowledge, skills and confidence of patients to manage their health,		1			
Patient Compentency Rating Scale (PCRS)	To assess self-awareness following brain injury.		1			
Paykel Suicide Scale	To measure or screen suicidal behavior in adolescents.		1			
Pearlin & Schooner Mastery	The Pearlin Mastery (PM) scale measures the extent to which an individual regards their life chances as being under their personal control rather than fatalistically ruled		1			
PEG-3 Item Pain Scale	A three-item scale assessing pain intensity and interference.			1		
PELI-NH	To identify nursing home residents' authentic desires.		1			
Penn State Worry Questionnaire (PSWQ)	To identify pathological worry in patients		1			
perceived social support (Interpersonal Support Evaluation List 12)	A total score that describes overall perceived social support, and three subscales representing perceived availability of appraisal (advice or guidance), belonging (empathy, acceptance, concern), and tangible (help or assistance, such as material or financial aid) social support.		1			
Perceived Stress Scale	Psychological functioning (incl. coping) - To assess the respondent's perception of stress (4,10,14 item)		1			
Person-centred community care inventory (PERCCI)	Quality of care experiences				1	
Personal Outcomes Scale	To measure the quality of life of individuals with learning disabilities.				1	
Personal Wellbeing Index - Intellectual Disability (PWI-ID)	A state-of-the-art instrument to measure subjective wellbeing in this population.		1			
PFSSADI	Assessing older adults' preferences for pain-related social support for functional autonomy or dependence could contribute to increase formal social support responsiveness to individuals' needs.		1			

PRO Measure	Construct	Functional Status	Psychological Status	Symptom Burden	Quality of Life	Other
Philadelphia Geriatric Center Morale Scale (PGCMS)	To evaluate three factors: Agitation, Attitude Toward Own Aging, and Lonely Unsatisfaction		1			
PHQ-9	To measure depression severity and to diagnose depressive disorders		1			
Pittsburgh Sleep Quality Index (PSQI)	To provide a reliable, valid, and standardized measure of sleep quality, to discriminate between "good" and "poor" sleepers, to provide an index that is easy for subjects to use and for clinicians and researchers to interpret, to provide a brief, clinically useful assessment of a variety of sleep disturbances that might affect sleep quality	1				
Post-discharge Coping Difficulty Scale (PDCDS)	Measuring difficulty with coping at home following discharge from the hospital.		1			
Process of Personal Recovery (Neil 2 9)	To assess the process of recovery - To develop and validate a short recovery questionnaire in collaboration with service users.		1			
Profile of Anger Coping Skills	An evaluation instrument designed to assess an individual's usage of specific anger coping skills.		1			
PROMIS	Different measures of social role performance and participation.			1		
Provocation Index	Identifies the kind of situations that induce anger in particular individuals.		1			
PTSD symptoms (Primary Care Post-Traumatic Stress Disorder screener, PC-PTSD)	To assess whether the respondent has had any exposure to traumatic events.		1			
QoL in Alzheimer's Disease (QOL-AD)	To develop and provide psychometric data on perceived QoL in AD patients as rated by patients and caregivers				1	
QoL questionnaire	To determine respondents general quality of life, their natural environment, their health, their living condition, their community and more.				1	
QOL Scales for Nursing Home Residents	A scale aiming to assess the QOL of nursing home residents.				1	
Quality of Life after Traumatic Brain Injury (QoLIBRI)	To assess health-related quality of life (HRQoL) of individuals after traumatic brain injury.				1	
Quality of Life GRS	Aims to assess the QOL in transwomen following Gender reassignment surgery (GRS).				1	

PRO Measure	Construct	Functional Status	Psychological Status	Symptom Burden	Quality of Life	Other
Quality of Life in Life-Threatening Illness-Family Carer Version (QOLLTI-F)	To measure multiple dimensions and overall quality of life of the family caregivers of people with a life-threatening illness.				1	
Quality of Life Index (QoLI-2)	A quality of life measure developed for use in people with mental illness, which assesses satisfaction with family relationships, social relationships, finances, leisure, living situation, safety, and overall quality of life				1	
Rating Anxiety in Dementia (RAID)	A clinical rating scale developed to evaluate anxiety in persons with dementia.		1			
Resilence Scale (RS)	To identify the degree of individual resilience		1			
Rockwood Clinical Frailty Scale	Evaluates specific domains including comorbidity, function, and cognition to generate a frailty score ranging from 1 (very fit) to 9 (terminally ill).	1				
Rosenberg Self-Esteem Scale (RSES)	To assess self-esteem in the community		1			
Satisfaction with Life Scale	Patient satisfaction (global judgment of one's life rather than satisfaction with specific domains) - To assess global life satisfaction		1			
Scale for Suicidal Ideation	To assess the severity or intensity of suicidal ideation		1			
Self-<br>Rated Health Question (SRH; Idler & Benyamini, 1997)	Measures of mental and physical health status	1				
Self-efficacy for Managing Chronic Disease scale	To measure self-efficacy for patients with systemic sclerosis		1			
Self-Management Ability Scale (SMAS-3)	To measure self-management abilities (SMA) - 3 item		1			
Self-perceived health (Robine)	Assesses ability to perform activities of daily living (ADLs) and instrumental activities of daily living (IADLs).	1				
Self-reported quality of life scale	Focuses on the subjective domain and aims to allow mental health service users to express their own views about their lives.				1	
Sense of Direction and Spatial Representation Questionnaire (SDSRQ)	The SDSR consisted of 13 items measuring SOD, spatial representation,<br>and orientation strategies.		1			
Sense of security in Care instrument for patients	Measuring the patients' and relatives' sense of security in palliative care.		1			

PRO Measure	Construct	Functional Status	Psychological Status	Symptom Burden	Quality of Life	Other
SF-12	Developed to be a much shorter, yet valid, alternative to the SF-36® for use in large surveys of general and specific populations as well as large longitudinal studies of health outcomes				1	
SF-36	The SF-36 was developed during the Medical Outcomes Study (MOS) to measure generic health concepts relevant across age, disease, and treatment groups				1	
Sheldon-Cohen Perceived Stress Scale	To assess the respondent's perception of stress (4,10,14 item)		1			
Short Falls Efficacy Scale	To measure the fear of falling in older adults while performing social and physical activities, both inside and outside the home	1				
Short Physical Performance Battery (SPPB)	To assess lower extremity function	1				
Single item on Loneliness	A measure of degree of loneliness on scales ranging from never to always.		1			
Single item QoL rating	These single items measured physical, emotional, and social health, pain, overall health, and quality of life.				1	
Smile-o-meter	Used in adults with cognitive and physical disabilities 33 and consists of a self-reported scale of mood based on a 5-point Likert-type scale - Not sure what it assesses, smiles??		1			
Social Identification and Satisfaction (SIS)	Measuring social isolation, relations and acceptance.		1			
Social Support Inventory	assess satisfaction with obtained social support using Simultaneous Components Analysis (SCA) is described. In the first study the com		1			
Social Support Questionnaire	A measure of social support - overall level of satisfaction with the support given in each of area.		1			
Social Support Scale of Medical Outcomes Study (SSS-MOS)	Measures the availability of support, if needed, in several domains.		1			
Spatial Anxiety Scale (SA)	A measure of spatial anxiety for adults.		1			
Spatial Self-Efficacy Questionnaire (SSEQ)	Measures stroke survivors' self-efficacy with self-management and activities as two unidimensional constructs.		1			
SSRS (measure degree of social support)	The SSRS offers several unique features to facilitate more comprehensive assessment and intervention services for children experiencing social behavior problems. It is the first social skills		1			

PRO Measure	Construct	Functional Status	Psychological Status	Symptom Burden	Quality of Life	Other
	rating scale to provide norms based on a large, national sample of boys and girls ages 3 through 18					
Substance disorder screener	To determine the level of impact substance use is having on the individual's health both physical and mental		1			
Suicide Risk Assessment	Measures four different areas of suicide risk.		1			
Symptom Bother Scale	Measures the frequency of urgency, nocturia, and incontinence symptoms. (not too sure about this, seems out of place)			1		
The Alcohol, Smoking, and Substance Involvement Screening Test (ASSIST)	The aim is to detect psychoactive substance use and related problems among primary care patients.		1			
The Attitude to Aging Questionnaire (AAQ)	The subjective perception of ageing.		1			
UCLA Loneliness Scale	To measure loneliness.		1			
UCLA Social Support Inventory	Measures an individual's perceived social support. It assesses the perceived availability and satisfaction with social support from various sources, including family, friends, and significant others which can have an impact on an individual's overall wellbeing, coping mechanisms and psychological adjustment.		1			
Urban life stress (Urban Life Stress Scale, ULSS)	To measure community-level stressors as potential sources of psychological and emotional stress experienced by persons living in medium to large cities in 3 domains (economic stability, social & community context, and neighbourhood & physical environment).		1			
Utrecht Symptom Diary-4 Dimensional	An instrument that can be used to monitor symptoms and needs in the physical, psychological, social, and spiritual dimensions and to optimize communication between patients and caregivers.			1		
VAS (health status)	Overall health	1				
Visual Analogue Scale (VAS)	A unidimensional measure of pain intensity	1				
Warwick-Edinburgh Mental Health and Wellbeing Measure	To measure mental wellbeing		1			
WEll-being Star	To measure progress in living as well as possible for people living with a long-term health condition		1			
WHO-5	To measure positive psychological well-being				1	

PRO Measure	Construct	Functional Status	Psychological Status	Symptom Burden	Quality of Life	Other
WHODAS	To help identify needs, match patients to interventions, track functioning over time, measure clinical outcomes and treatment effectiveness	1				
WHOQOL	Measuring Quality of Life by The World Health Organization				1	
Wong Baker Pain Rating Scale (WBPRS)	Assessment of pain			1		
**Total Number of PROMs per classification**		**35**	**121**	**15**	**36**	**9**
